# Neutrophil swarming delays the growth of clusters of pathogenic fungi

**DOI:** 10.1038/s41467-020-15834-4

**Published:** 2020-04-27

**Authors:** Hopke Alex, Allison Scherer, Samantha Kreuzburg, Michael S. Abers, Christa S. Zerbe, Mary C. Dinauer, Michael K. Mansour, Daniel Irimia

**Affiliations:** 1 BioMEMS Resource Center, Massachusetts General Hospital, Boston, MA 02129 USA; 2 Harvard Medical School, Boston, MA 02115 USA; 3 Shriners Hospital for Children, Boston, MA 02114 USA; 4 Division of Infectious Diseases, Massachusetts General Hospital, Boston, MA 02114 USA; 5 Laboratory of Clinical Immunology and Microbiology (LCIM), National Institute of Allergy and Infectious Diseases (NIAID), National Institutes of Health (NIH), Bethesda, MD 20814 USA; 6 Departments of Pediatrics and of Pathology and Immunology, Washington University School of Medicine in St. Louis, St. Louis, MO 63110 USA

**Keywords:** Infectious diseases, Innate immune cells, Fungal host response

## Abstract

Neutrophils employ several mechanisms to restrict fungi, including the action of enzymes such as myeloperoxidase (MPO) or NADPH oxidase, and the release of neutrophil extracellular traps (NETs). Moreover, they cooperate, forming “swarms” to attack fungi that are larger than individual neutrophils. Here, we designed an assay for studying how these mechanisms work together and contribute to neutrophil's ability to contain clusters of live *Candida*. We find that neutrophil swarming over *Candida* clusters delays germination through the action of MPO and NADPH oxidase, and restricts fungal growth through NET release within the swarm. In comparison with neutrophils from healthy subjects, those from patients with chronic granulomatous disease produce larger swarms against *Candida*, but their release of NETs is delayed, resulting in impaired control of fungal growth. We also show that granulocyte colony-stimulating factors (GCSF and GM-CSF) enhance swarming and neutrophil ability to restrict fungal growth, even during treatment with chemical inhibitors that disrupt neutrophil function.

## Abstract (web summary)

Neutrophils employ several mechanisms to control the growth of fungi, including enzymes, reactive oxygen species, extracellular traps, and formation of “swarms”. Here, Hopke et al. study how the different mechanisms work together, using an in vitro assay with human neutrophils and clusters of live *Candida* cells.

## Introduction

Neutrophils represent a key effector in the innate immune system and are critical for defense against microbes, especially invasive fungal infections^1,2^. However, neutrophils are also highly destructive and are involved in the pathology of numerous inflammatory conditions^3–5^. Manipulation of neutrophil function, therefore, represents an attractive avenue that could benefit patients during infections or during inflammation. Unfortunately, significant gaps in our understanding of neutrophil behavior represent a major obstacle which blocks our ability to design therapies that could selectively enhance or dampen neutrophil function in different contexts, as appropriate. As an example of this knowledge gap, neutrophil swarming, a novel aspect of neutrophil behavior by which human neutrophils coordinate their recruitment against large clusters of microbes, has only recently been uncovered^6,7^. During swarming, neutrophils cooperate and enhance their antimicrobial activities beyond the simple sum of a similar number of independent neutrophils. Swarming is also a selective behavior, triggered by targets larger than a threshold size, and distinct from phagocytosis^8^. While it is thought that swarming represents an important mechanism in the response to infections, the molecular mechanisms contributing to microbe control are poorly defined. The cooperation between human neutrophils is mediated by a constellation of mediators that direct the neutrophils toward the target and enhance their antimicrobial activities in a chain-reaction manner. In vitro studies have identified that neutrophils can produce their own stop signal that disrupts the chain reaction and brings the swarm to a dynamic equilibrium, though more signals likely play a role in coordinating this process in vivo^8^. The role of swarming is context dependent, as it plays supporting roles in host defense against some pathogens^9–13^, but is detrimental against others^14–18^. Thus a more detailed understanding of the interactions between neutrophil swarms and microbes will complement the current paradigm of one neutrophil–one microbe interaction, when microbes are phagocytosed and destroyed inside individual neutrophils. That said, significant methodological challenges must be overcome to examine the mechanism of swarming, because it is currently slow and difficult to rigorously test swarming with in vivo models. Current in vitro models for studying phagocytosis cannot be directly extrapolated to swarming.

Here we design and validate microscale technologies to analyze human neutrophil swarming behavior against live fungi. We demonstrate that human neutrophils swarm against clusters of live fungal pathogens such as *Candida* *albicans*. We show that swarming contributes to the containment of *C*. *albicans* and the process involves the action of LTB_4_, myeloperoxidase (MPO), reactive oxygen species (ROS), and neutrophil extracellular traps (NETs). While many of these molecules are also involved in traditional one-to-one host–pathogen interactions, their roles in the context of swarming against live microbe clusters are not understood. We show that ROS, for example, can function as both an antimicrobial factor as well as a determinant of swarming dynamics. Importantly, we show that neutrophil swarming function can be enhanced by the addition of granulocyte macrophage colony-stimulating factor (GM-CSF) or GCSF. This enhancement can also be used to rescue antifungal activity during conditions where swarming would otherwise be defective. Taken together, these results demonstrate that this assay will be an effective tool for the molecular dissection of human neutrophil swarming, a screen for mediators that impact neutrophil function and may ultimately contribute to improving therapeutic designs for infections.

## Results

### Live fungi arrays for interrogation of human neutrophil swarming

We monitored the interactions between human neutrophils and clusters of live microbes using a versatile platform that can accommodate various fungi. The platform arranges microbes in clusters of 100-µm diameter, grouped in 8 × 8 arrays, separated inside individual wells, in a 16-well format. Using this platform, we successfully patterned numerous live fungal targets, including *C*. *albicans*, *Candida* *glabrata*, and *Candida* *auris*, as well as *Aspergillus fumigatus* (Supplementary Fig. 1). All the microbes tested grew well on the patterns, and *C*. *albicans* and *A*. *fumigatus* were both able to form robust hyphae (Supplementary Fig. 1, Supplementary Movie 1).

### Human neutrophil swarming restricts fungal growth

**Fig. 1:**
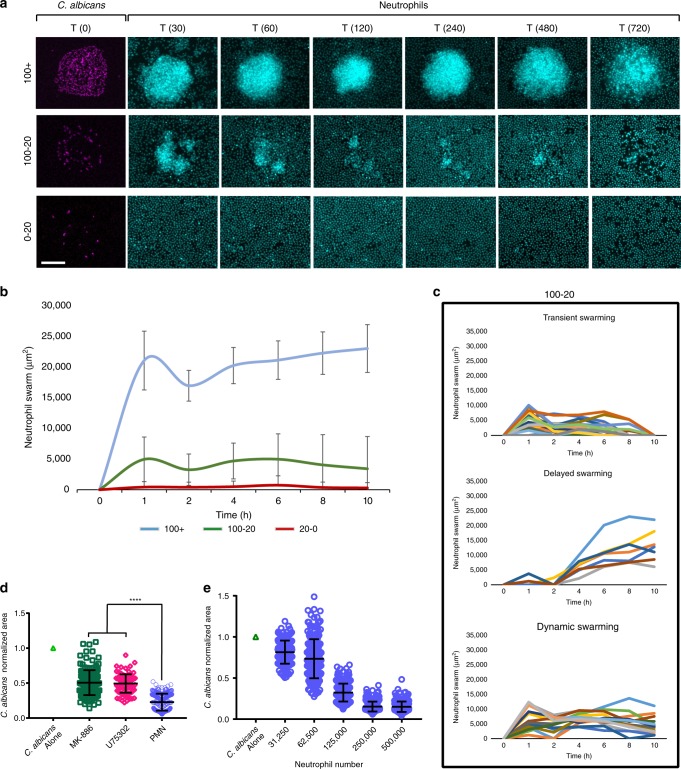
Neutrophil cooperation against clusters of *C. albicans*. **a** Living *C. albicans* were patterned in clusters on poly-l-lysine/zetag arrays at different densities. Purified human neutrophils were added to the arrays to observe host-pathogen interactions. Time-lapse images show the sustained neutrophil swarming to large clusters (100+ yeast), a mix of dynamic swarm sizes over time during the swarming responses to intermediate sized clusters of yeast (100-20) and no swarming to scattered yeast. **b** The dynamics of swarming responses, measured by area of the swarm, was quantified over time. Lines show the average of the responses for each group. **c** N = 24 swarms for the 100+ group, 42 for the 100-20 group and 29 for the 20-0 group across two independent donors. Individual swarm tracks are shown for the 100-20 group, split by qualitative phenotype. **d** Swarming was conducted in the presence of the LTB_4_ synthesis inhibitor (MK-886 1 µM) or blockers of the LTB_4_ receptor BLT1 (U75302). The area of *C. albicans* growth was quantified at 16 hours. Results are normalized to the growth of the *C. albican*s alone control. The results of untreated neutrophils are shown as a baseline. N = 144 swarms across 3 donors for MK-886, 96 swarms from one representative donor for U75302 and 240 swarms across three donors for the untreated PMN. **e** Different numbers of human neutrophils were incubated with arrays of *C. albicans* and fungal growth was quantified at 16 hours. Results were normalized to the growth of the *C. albicans* alone control. N = 192 swarms across three different donors. Error bars represent mean +/− standard deviation. ****p< 0.0001 Kruskal-Wallis with Dunns post-test. Scale bar is 50 µm.

Upon the addition of human neutrophils to these arrays, we achieved robust, synchronized swarming responses against clusters of live fungi. We observed rapid, exponential recruitment of neutrophils to the targets within the first 30 min followed later by a plateau (Fig. 1a, b). This sustained swarming was a selective response to large *C*. *albicans* clusters (Fig. 1a, b, Supplementary Fig. 2). Smaller clusters of yeast (20–100 cells) could trigger a range of dynamic behaviors (Fig. 1a–c, Supplementary Fig. 2). These included “transient swarms” that had resolved on their own by the end of the assay, “dynamic swarms” that fluctuated rapidly but were not resolved by the end of the assay, and delayed swarms that triggered later in the assay (Fig. 1c). Small numbers of scattered yeast (0–20) did not trigger swarming, again demonstrating that there is discrimination between situations where phagocytosis or swarming is appropriate (Fig. 1a, b, Supplementary Fig. 2). The exponential recruitment of neutrophils and swarming to *C*. *albicans* clusters is similar to that described against zymosan targets and suggestive of the positive feedback loops mediated by neutrophils and neutrophil-released mediators typical for swarming^8^. Disruption of LTB_4_, a key mediator for swarming responses, by inhibition of LTB_4_ synthesis with MK886 or blocking the LTB_4_ receptor BLT1 with U75302 both resulted in partial loss of control of *C*. *albicans* growth (Fig. 1d). These defects were only partial though, in keeping with the redundant nature found previously in human swarming mediators^8^. Finally, the efficacy of swarming at containing microbe growth depends on the number of neutrophils available for swarming at one location. The dependence is represented by a sigmoid curve rather than a line, suggesting a threshold effect for fungal control during swarming (Fig. 1e). Taken together, these data demonstrate that clusters of live *C*. *albicans* can trigger neutrophil swarming, a stronger and more complex response than the simple accumulation of neutrophils on sparse targets. The dynamics of this response are influenced by both the microbial target and the neutrophils themselves. We also showed that this process was capable of restricting fungal growth. We therefore leveraged this assay to characterize the dynamics and molecular mechanisms by which swarming restricts *C*. *albicans* and other fungi.

**Fig. 2:**
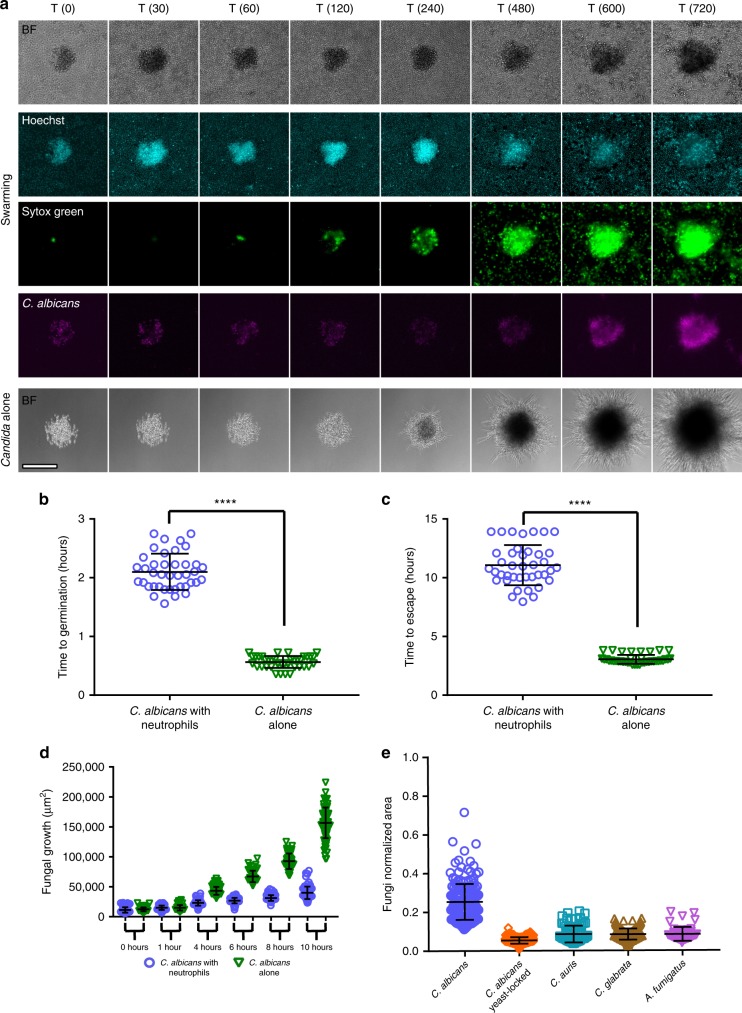
Neutrophil swarming delays germination and restricts the growth of *C. albicans* and other fungi. **a** Time-lapse images show the progression of neutrophil swarming to *C. albicans* patterns. Brightfield, DAPI (Hoechst staining), FITC (Sytox green) and Cy5 (*C .albicans*) channels are presented. **b** Representative panels of *C. albicans* growing alone is shown as a comparison (a, fifth row). *C. albicans* germination is delayed by swarming. N = 40 swarms across 3 donors. **c** *C. albicans* escape from a defined area (equivalent to the area of the swarm) is significantly delayed by neutrophils. N = 40 swarms across 3 donors. **d** The area of *C. albicans* pattern remains smaller in the presence of neutrophils, showing that human neutrophil swarming restricts fungal growth. N = 104 swarms across three different donors for all timepoints except 6 and 8 hours, where N = 72 swarms across three different donors. **e** Different fungal species were incubated with and without human neutrophils and fungal growth was quantified at 16 hours. Results were normalized to the growth of the respective fungi alone control. N = 182 swarms for the *C. albicans* group, N = 192 swarms for the *C. albicans* yeast-lockedgroup, N = 73 swarms for the *C. auris* group and N = 199 for the *C. glabrata* group, all across three independent donors. For *A. fumigatus*, N = 48 swarms with one donor. Quantification of the area of fungal growth during incubation of the *C. glabrata* or *C. auris* with or without neutrophils over time, showing that human neutrophil swarming restricts the growth of these fungi in (**d**-**e**). N = 45 swarms across three different donors for *C. glabrata* in (**d**). N≥16 swarms across two different donors for *C. auris* in (**e**). ****p <0.0001 Students T-test (unpaired, two-tailed). Error bars represent mean +/− standard deviation. Scale bar is 100 µm.

The growth of *C*. *albicans* was restricted by neutrophil swarms at multiple points. First, *C*. *albicans* germination from yeast to hyphae was significantly delayed during neutrophil swarming (Fig. 2a, b). By comparison, *C*. *albicans* patterned on arrays in the absence of neutrophils starts germinating within 30 min (Fig. 2a, b). Even when surviving, hyphae from germinating *C*. *albicans* are growth restricted by neutrophil swarming for ≥10 h, requiring on average nearly 11 h to eventually penetrate the swarm and escape containment (Fig. 2c). At 10 h, colonies attacked by swarming were approximately three times smaller than *C*. *albicans* allowed to grow without neutrophils, showing the significant restriction that swarming can exert on fungal growth (Fig. 2a–d). We also found that swarming significantly contains the growth of clusters of other fungi, like *C*. *glabrata*, *C*. *auris*, and *A*. *fumigatus* (Fig. 2e, Supplementary Fig. 3). The swarms formed against *C*. *auris* and *C*. *glabrata* ended up smaller than those seen against *C*. *albicans*, despite the initial clusters being of identical size, demonstrating that the dynamics and magnitude of swarming responses can be dictated by the fungal species being engaged (Fig. 2c–e). Interestingly, *C*. *auris* and *C*. *glabrata* do not produce hyphae, while *C*. *albicans*, which induces larger swarms, can switch from yeast to hyphae. To directly probe the impact of this transition to hyphae in the swarm/fungi interaction, we tested a yeast-locked mutant of *C*. *albicans* on the swarming array. We found that the yeast-locked mutant resulted in less robust swarming responses and NET release than the wild-type *C*. *albicans*, which could make hyphae (Fig. 2e, Supplementary Fig. 3). We also found that the yeast-locked mutant was more effectively cleared than the wild-type *C*. *albicans*, suggesting that hyphae play a critical role in mediating escape from containment by neutrophil swarming (Supplementary Fig. 3b).

### Neutrophils release NETs within swarms that restrict fungi

**Fig. 3:**
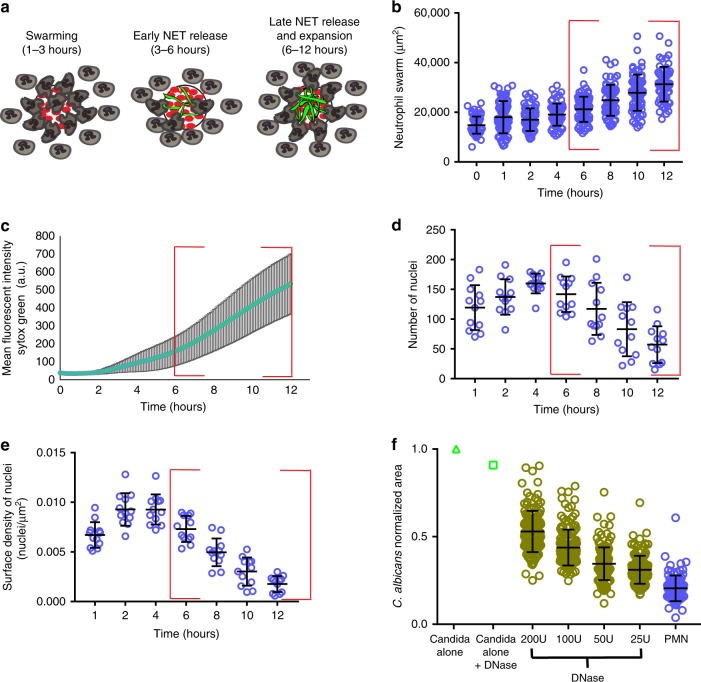
Human neutrophils release NETs during swarming to *C. albicans*. Human neutrophils were incubated with *C. albicans* arrays with Sytox green included in the solution to visualize NET formation during swarming. A cartoon model is shown in (**a**), demonstrating the distinct phases of swarming and NET release. The area of the neutrophil swarms around *C. albicans* (**b**), the mean fluorescent intensity of Sytox green staining in the swarm (**c**), the number of nuclei in the swarm (**d**) and the number of nuclei/µm^2^ (**e**) were all quantified. A red box highlights the timeframe of NET formation and late phase swarm expansion (**b**–**e**). N = 68 swarms across three donors for except for the 12 hour timepoint, where N = 60 swarms across three donors (**b**). N = 44 swarms for (**c**). N = 12 swarms for **d** and **e**. The inclusion of DNase in the media during the swarming assay results in a dose dependent loss of control of *C. albicans* growth (**f**). Results are normalized to the growth of the *C. albicans* alone control. N = 144 swarms over 3 independent donors except for the control PMN group where N = 95 swarms over 3 donors. Error bars represent mean +/− standard deviation.

Following the initial swarm growth and plateau, we found that the area of the neutrophil swarms against *C*. *albicans* clusters continued to expand ~8 h after the start of the assay (Figs. 2 and 3). This expansion was not accompanied by significant further neutrophil recruitment, which ended by ~4 h. Impressively, this expansion coincided with the release of NETs by the neutrophils engaged in the swarm (Figs. 2a, b and 3a–e). NET release was quantified by examining the appearance of Sytox green staining within the swarm over time (Fig. 3c, Supplementary Movie 1). The release of NETs against clusters of live *C*. *albicans* occurred in 100% of the swarms and was synchronous. We also quantified the number of visible, condensed neutrophil nuclei within the swarm over time (Fig. 3d, e). Sytox green staining intensity and the loss of visible nuclei showed inverse trends, supporting the release of NETs within swarms (Fig. 3b–e). These NETs also stained positive for the additional NET markers, including citrullinated histone H3 (Supplementary Fig. 4).

The release of NETs was important for the continued restriction of *C*. *albicans* growth. The inclusion of DNase in the assay resulted in degradation of NETs and compromised the ability of swarms to control fungal growth (Fig. 3f). Inclusion of DNase reduced the observed Sytox green staining to nearly negligible levels, demonstrating it was indeed degrading the NETs (Supplementary Fig. 5). Treatment with DNase also led to a significant reduction in the expansion of swarms at later time points (i.e., 8 and 10 h), further supporting that NET release helps drive the expansion of the swarm. Interestingly, the DNase-treated swarms did still show smaller levels of expansion at later time points. This suggests that other factors, like the growth of surviving *C*. *albicans* hyphae within the swarm, contribute to swarm expansion. Indeed, the growth of surviving *C*. *albicans* within the swarm also closely lines up with overall swarm area expansion (Figs. 2a, d and 3, Supplementary Fig. 5). To further dissect swarm expansion at later time points and establish whether NET release during swarming is specific to interactions with *C*. *albicans*, we patterned commercially available bioparticles derived from *Staphylococcus* *aureus* and *Escherichia* *coli* (Supplementary Fig. 6). These particles are fluorescently labeled and gave excellent coverage of the printed arrays. We observed synchronized NET release against every target in the *S*. *aureus* and *E*. *coli* particle arrays. These observations show that expansion of neutrophil swarms at later time points is not unique to just live *C*. *albicans* and coincides with the release of NETs for all targets tested (Fig. 3, Supplementary Fig. 6). Interestingly, the magnitude of the swarms was different against different pathogens. Neutrophil swarms against *S*. *aureus* particle clusters were larger than against *E*. *coli* particle clusters of identical dimensions (Supplementary Fig. 6c). This result, along with the comparison of swarming to different fungal species shown above, suggests that swarming dynamics can be influenced by the target (Figs. 2 and 3, Supplementary Figs. 3–6).

### ROS and MPO regulate swarm dynamics and fungal restriction

**Fig. 4:**
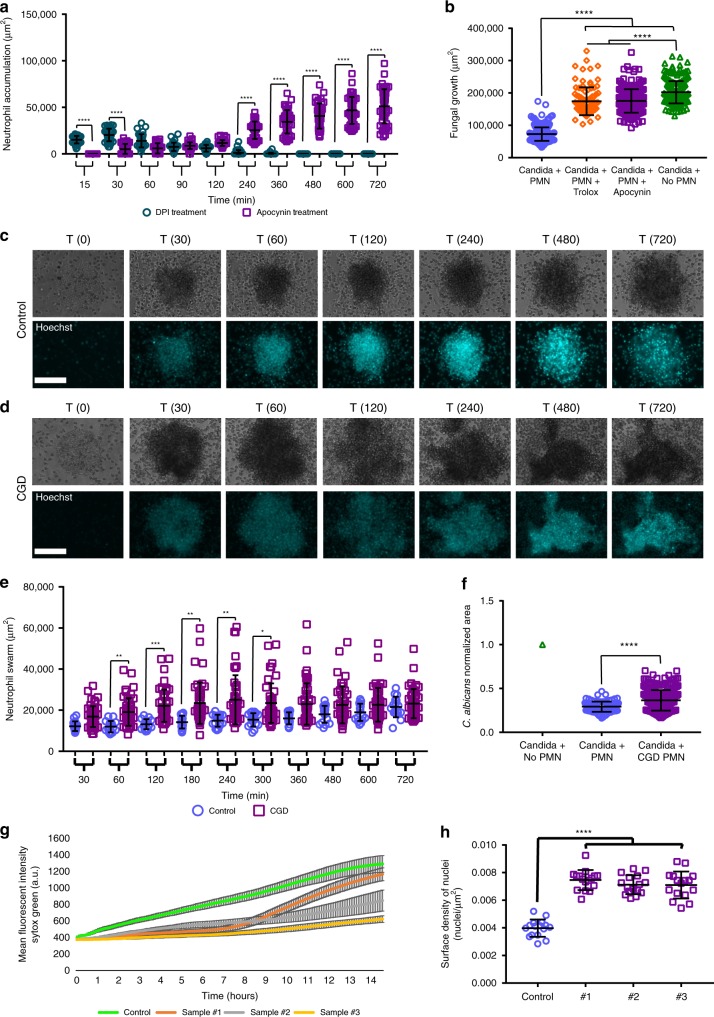
NADPH oxidase function plays a critical role in swarm dynamics. Human neutrophils were incubated with different inhibitors to interrogate the mechanisms necessary to restrict the growth of *C. albicans* during swarming. The inclusion of 300 µM Apocynin disrupted the ability of neutrophils to swarm to *C. albicans*, while the inclusion of 10 µM DPI resulted in rapid formation and then dissolution of swarms, as shown in quantification of the neutrophil accumulation area. N = 36 swarms across 3 donors for DPI, N = 48 across 3 donors for Apocynin (**a**). The area of fungal growth after swarming with ROS inhibitors at 16 hours shown (**b**). N = 288 swarms for PMN, 96 swarms for Trolox, 286 swarms for Apocynin and 219 spots for the no PMN group, across 3 independent donors. CGD neutrophils exhibit exaggerated swarming at early points in the assay as shown in a panel of representative images and in the quantification of neurophil swarming area (**c**–**e**). N = 16 swarms for one donor for control and N = 46 swarms across three donors for CGD. The area of fungal growth after swarming with control or CGD neutrophils after 16 hours is shown (**f**). Results are normalized to growth of the *C. albicans* alone control. N = 96 swarms for one donor for control and N = 288 swarms across three donors for CGD. CGD samples exhibit reduced NETs. The MFI of Sytox Green staining was quantified for swarms from a healthy donor and from CGD neutrophils (**g**). Average of 16 swarms for control and 16 swarms for each individual CGD sample is shown. The number of nuclei/µm^2^ was quantified and is shown (**h**). N = 16 swarms for one donor for control and N = 16 swarms for each individual CGD sample.*p ≤ 0.05 (*p* = 0.0489 in **e**), **p ≤ 0.01 (*p* = 0.0068 for control 60 vs CGD 60, *p* = 0.0011 for control 180 vs CGD 180, *p* = 0.0030 for control 240 vs CGD 240 in **e**), ***p ≤ 0.001 (*p*=0.0001 for control 120 vs CGD 120 in **e**), ****p <0.0001 (**a, b, f, h**). Kuskal-Wallis with Dunn’s post-test for **a**-**b**, **e**, **h**; Mann-Whitney for **f** (unpaired, two-tailed). Error bars represent mean +/− standard deviation. Scale bar is 100 µm.

As outlined above, we can leverage our assay to better understand the molecular mechanisms of microbial containment, and we found that NETs were critical for the containment of *C*. *albicans* hyphae. Probing further into the molecular mechanisms, we also found that swarming and containment of *C*. *albicans* involved NADPH oxidase and MPO function. In the presence of an NADPH oxidase inhibitor, Apocynin, swarming was inhibited early, and neutrophils responded at later points in the assay, as the *C*. *albicans* hyphae grew outwards (Fig. 4a, Supplementary Fig. 7, Supplementary Movie 2). We designated this analysis “area of accumulation,”  as Apocynin-treated neutrophils did eventually recruit to the fungi, but they usually did not display the rapid, exponential recruitment characteristic of true swarming. Inhibition by Apocynin reduced the ability of neutrophils to restrict *C*. *albicans*, resulting in significantly faster germination and escape from neutrophil containment, >6 h earlier than in the control (Supplementary Fig. 7). We did see the release of NETs in the presence of Apocynin, though they were clearly insufficient to restrict fungal growth in this context. To further test the role of reactive oxygen, we also tested the effect of the presence of antioxidant Trolox. While swarming occurred in the presence of Trolox, there was also a defect in the ability to restrict *C*. *albicans* growth (Supplementary Fig. 7e, f). However, this defect appeared much later than the defect seen during Apocynin treatment (Supplementary Fig. 7e). In the presence of diphenyleneiodonium (DPI), another classic NADPH oxidase inhibitor, neutrophil swarms formed rapidly; however, they also quickly disintegrated, which stands in contrast to the phenotype observed during Apocynin treatment (Fig. 4a, Supplementary Fig. 7a, b). We were unable to determine the impact of DPI on fungal control, as the DPI also directly impacted fungal growth, something that was not observed with other inhibitors (Supplementary Fig. 7e, f). In addition, we did not see the formation of NETs in the transient swarms formed during DPI treatment (data not shown).

As chemical inhibitors, Apocynin and DPI are known to have off-target impacts, so we generated a p47 (NCF1)-deficient neutrophil cell line using a Cas9-ER-HoxB8 mouse cell line (Supplementary Figs. 8 and 9). This line was confirmed to be deficient in NCF1 by western blot and to be severely deficient in ROS production during interactions with fungi (Supplementary Fig. 8). These neutrophils displayed a very different phenotype from the chemical inhibitors, with enhanced swarming responses, especially increased recruitment, compared to the control (Supplementary Fig. 8a, b). Despite this increased recruitment, restriction of *C*. *albicans* growth was still significantly compromised, confirming an antimicrobial role of ROS in this system (Supplementary Fig. 9c). Importantly, the control cells showed a similar phenotype to human neutrophils when treated with Apocynin, suggesting that the delayed swarming phenotype is not unique to human cells and is due to off-target effects. To confirm this observation, the NCF1-deficient cells were treated with Apocynin. Interestingly, we found that the knockout (KO) cells treated with Apocynin showed no significant changes; however, it is thought that, as Apocynin is activated by a process involving oxidation and without any ROS production, the NCF1-deficient cells are unable to activate the drug, thereby avoiding off-target impacts from manifesting (Supplementary Fig. 9f)^19^. To fully resolve this conflict of phenotypes, we obtained neutrophils from humans with chronic granulomatous disease (CGD), which have specific mutations that render their NADPH oxidase deficient in ROS production. The results from these CGD neutrophils aligned with the results from mouse NCF1-deficient cells, with human CGD neutrophils showing significantly larger neutrophil swarms for up to the first 5 h of swarming against live *C*. *albicans* (Fig. 4e, Supplementary Fig. 10). Human CGD cells also showed a relatively small but significant defect in *C*. *albicans* restriction (Fig. 4f). In keeping with the literature, CGD neutrophils also displayed reduced NET release compared to the healthy control (Fig. 4g, h, Supplementary Fig. 10d).

**Fig. 5:**
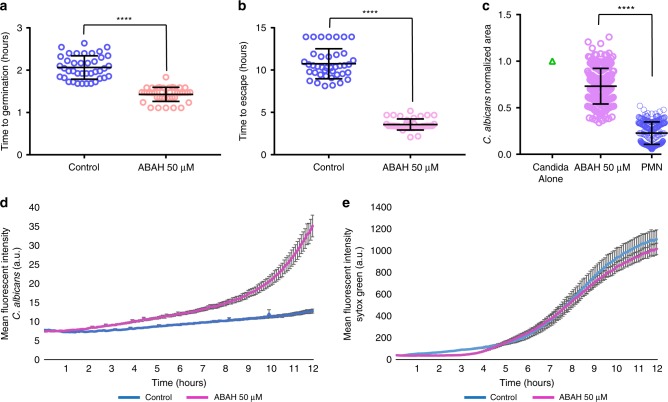
Myeloperoxidase function is important during swarming control of *C. albicans* germination and growth. *C. albicans* germinates faster in swarms treated with ABAH. N = 40 swarms across 3 donors (**a**). Fungal hyphae escape faster in swarms during MPO inhibition. N = 40 swarms across 3 donors (**b**). MPO inhibition results in more fungal growth at 16 hours. Results are normalized to the growth of the *C. albicans* alone control. N = 240 swarms over 3 donors (**c**). The MFI of *C. albicans* was quantified over 12 hours with and without MPO inhibition. N = 16 swarms from a representative donor (**d**). MPO inhibition does not impact NET release during swarming. The MFI of Sytox Green was quantified over 12 hours. N = 16 swarms from a representative donor (**e**). ****p <0.0001 Students T-test for **a–b**, two-tailed Mann-Whitney test for **c** (unpaired, two-tailed). Error bars represent mean +/− standard deviation for (**a**–**c**) and mean +/− standard error for (**d**–**e**).

We also inhibited MPO function using the chemical inhibitor ABAH and found that neutrophils were significantly inhibited in their ability to restrict fungal growth, with *C*. *albicans* again germinating faster and escaping swarming-mediated confinement >6 h earlier than in the controls (Fig. 5a–d). Interestingly, we noticed that NET release was not significantly different than the vehicle controls during MPO inhibition, suggesting that NET formation is not critically dependent on MPO in this context (Fig. 5e). These results suggest that both ROS and MPO contribute to the control of fungal growth, while ROS also seems to play a role in swarm dynamics.

### Cytokine treatment boosts swarming and rescues fungal restriction

**Fig. 6:**
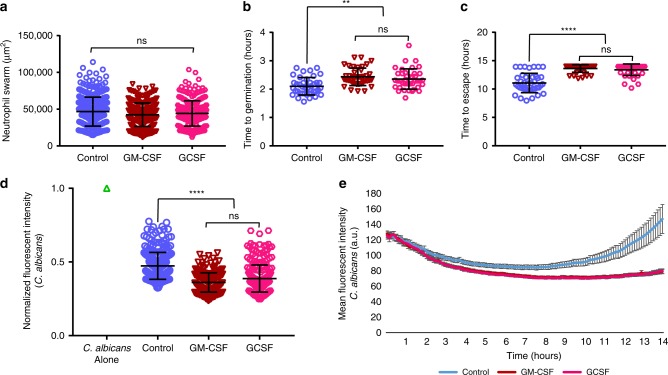
GM-CSF and GCSF enhance neutrophil swarming and fungal restriction. GM-CSF and GCSF enhance the restriction of *C. albicans* without significantly increasing swarm size. The area of swarms with or without GM-CSF or GCSF treatment at 16 hours was quantified and shown. N = 357 swarms for the Control group, N = 331 swarms for the GCSF group and N = 309 for the GM-CSF group, all across 4 independent donors (**a**). GM-CSF or GCSF treatment further enhanced the delay in *C. albicans* germination when compared to untreated swarming. N = 40 swarms across 3 donors (**b**). GM-CSF or GCSF enhanced hyphal restriction, delaying hyphal escape, often for the entire 14 hour assay. N = 40 swarms across 3 donors (**c**). GM-CSF and GCSF enhanced *C. albicans* clearance and control. The MFI of *C. albicans* within swarms at 16 hours was quantified and shown, normalized to the fluorescence of the *C. albicans* alone condition. N = 285 swarms for the Control group, N = 271 swarms for the GCSF group and N = 284 swarms for the GM-CSF group, all across 4 independent donors (**d**). The MFI of *C. albicans* in swarms with or without GM-CSF or GCSF treatment was quantified over 14 hours and is shown. N = 16 swarms from a representative donor (**e**). n.s. is non-significant, **p ≤0.01 (p= 0.003 control vs GCSF, *p* < 0.0001 for control vs GM-CSF) and ****p <0.0001 (**c**, **d**). Kruskal-Wallis with Dunns post-test. Error bars represent mean +/− standard deviation for (**a**–**d**) and mean +/− standard error for (**e**).

Fungal infections usually afflict those with compromised immune systems, so we leveraged our assay to examine whether we could enhance the ability of neutrophil swarming to restrict fungal growth. The addition of GM-CSF or GCSF significantly boosted the ability of human neutrophil swarms to contain and kill *C*. *albicans*. The time to germination of *C*. *albicans* was further delayed, as was the average time until hyphae escaped the swarm (Fig. 6a–e). In fact, >70% of swarms treated with GM-CSF and GCSF prevented escape by *C*. *albicans* for the entire 14-h assay (Fig. 6c). Analysis of neutrophil recruitment by Hoechst fluorescence shows that both GM-CSF and GCSF treatment result in enhanced swarming, though the overall size of the swarm is not significantly different from the control at the end of the assay (Fig. 6a, Supplementary Fig. 11a, b). GM-CSF and GCSF do not significantly alter NET release profiles. This suggests that GM-CSF and GCSF act on early but not late stages of swarming (Supplementary Fig. 11c, d). Both GM-CSF and GCSF appeared to be equally effective at the chosen doses, as there was no significant difference between the two treatments in the ability to restrict fungal growth and escape (Fig. 6).

**Fig. 7:**
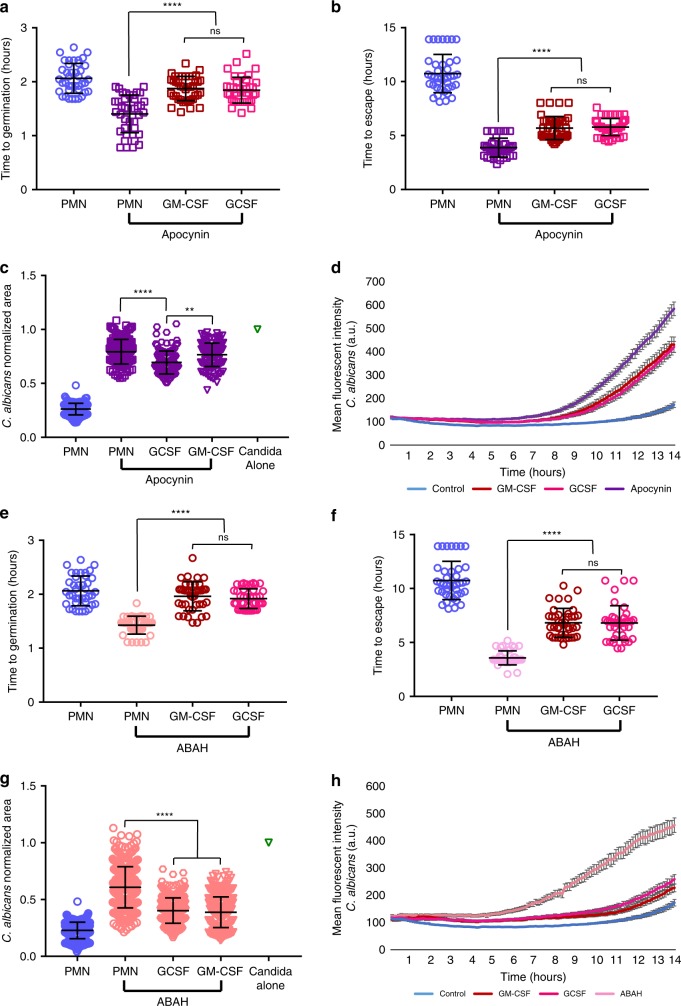
GM-CSF and GCSF treatment enhance swarming during ROS or MPO inhibition. GM-CSF or GCSF treatment can partially rescue swarming mediated fungal restriction. Apocynin treatment allows earlier *C. albicans* germination, while GM-CSF or GSF treatment restores some delay. N = 40 swarms across 3 donors (**a**). GM-CSF or GCSF treatment partially rescues swarming restriction of hyphal escape. N = 40 swarms across 3 donors (**b**). GM-CSF or GCSF treatment results in a small reduction in fungal growth at 16 hours. N = 256 swarms for the PMN group, N = 226 swarms for the Apocynin group, N = 210 swarms for the Apocynin+GCSF group, N = 208 swarms for the Apocynin+GM-CSF group, all across 3 independent donors (**c**). The MFI of *C. albicans* in swarms treated with Apocynin or both Apocyin and GM-CSF or GCSF was quantified over 14 hours. N = 16 swarms from a representative donor (**d**). ABAH treatment allows earlier *C. albicans* germination, while GM-CSF or GSF treatment restores some delay. N = 40 swarms across 3 donors (**e**). GM-CSF or GCSF treatment partially rescues swarming restriction of hyphal escape. N = 40 swarms across 3 donors (**f**). GM-CSF or GCSF treatment results in a reduction in fungal growth at 16 hours. N = 341 swarms in the PMN group, N = 355 swarms in the ABAH group, N = 342 swarms in the ABAH+GCSF group and N = 349 swarms in the ABAH+GM-CSF group, all across 3 donors (**g**). The MFI of C. albicans in swarms treated with ABAH or both ABAH and GM-CSF or GCSF was quantified over 14 hours. N = 16 swarms from a representative donor (**h**). n.s. is non-significant, **p = 0.0010 (**c**) and ****p <0.0001 (**a, b, c, e, f, g**). Kruskal-Wallis with Dunns post-test. Error bars represent mean +/− standard deviation for (**a**–**c**, **e**–**g**) and mean +/− standard error for (**d, h**).

**Fig. 8:**
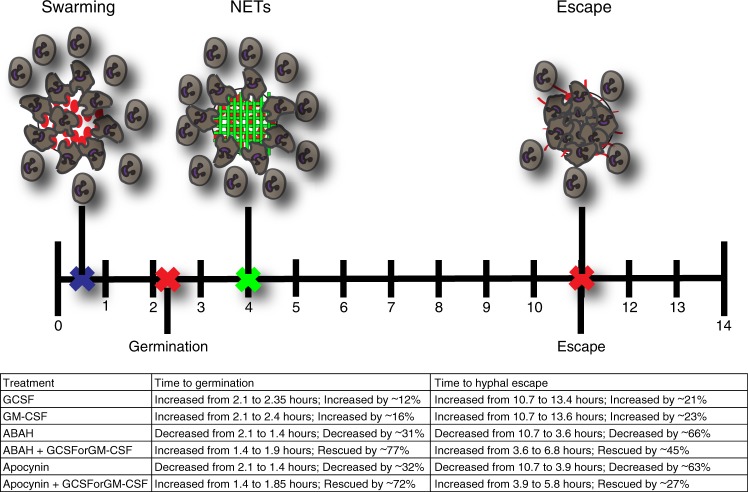
Summary of neutrophil swarming inhibition and enhancement. A timeline of the four major events in a 14-hour neutrophil swarming assay against *C. albicans*: neutrophil swarming, fungal germination, neutrophil NETs release, and hyphal escape. The average time of these events during swarming with healthy neutrophils is indicated with small “Xs” and cartoon depictions. The table summarizes the changes which occur to these events during treatment with different inhibitors (Apocynin and ABAH) and enhancers (GM-CSF and GCSF). Changes under the effect of inhibitors could be partially restored by the enhancers.

To further examine the interplay of molecular mechanisms by which swarming contains fungi and by which GM-CSF and GCSF enhance neutrophil swarming functions, we combined GM-CSF and GCSF treatment with the MPO inhibitor ABAH or the ROS inhibitor Apocynin. We found that both GM-CSF and GCSF treatment were able to enhance neutrophil swarming function during both ROS inhibition by Apocynin or MPO inhibition by ABAH, significantly rescuing the ability of swarming to delay *C*. *albicans* germination, block hyphal escape, and reduce overall fungal growth (Fig. 7a–h). In line with our findings above (Supplementary Fig. 6), GM-CSF and GCSF treatment enhanced neutrophil recruitment profiles during Apocynin or ABAH treatment but had minimal impact on NET profiles (Supplementary Fig. 11e–l). GM-CSF- or GCSF-mediated enhancement is more limited during ROS inhibition than the rescue seen for MPO, however, resulting in more fungal growth and faster hyphal escape despite GM-CSF and GCSF (Fig. 7). This observation remained consistent in Cas9-ER-HoxB8 control neutrophils treated with Apocynin as well (Supplementary Fig. 9d). Importantly, the ability of GM-CSF or GCSF to rescue function was also seen in the NCF1 KO cells, directly demonstrating that they can rescue some antifungal function in the absence of a functional NADPH oxidase (Supplementary Fig. 9). Together, these results identify GM-CSF and GCSF as cytokines that can directly boost the antifungal function of neutrophils (Fig. 8). This enhancement can proceed without MPO function and during Apocynin-mediated inhibition. Overall, we have optimized a device that allows for detailed and high-throughput examination of human neutrophil swarming against live fungi. We have leveraged this device to elucidate the molecular mechanisms required for swarming to restrict fungal growth, identifying NETs, MPO, and ROS as important players. We have also identified the cytokines GM-CSF and GCSF as mediators that can directly impact swarming to boost antifungal function, even when critical antifungal mechanisms are absent.

## Discussion

We designed and optimized an in vitro assay to facilitate the systematic analysis of human neutrophil interactions with clusters of growing *C*. *albicans*. The assay expands on the traditional paradigm of phagocytosis, when neutrophils act as independent agents against individual, planktonic microbes to reveal the several mechanisms through which human neutrophils work together to restrict the growth of clusters of pathogenic *C*. *albicans*. Consequently, this assay represents a significant advance from current in vitro assays of host–pathogen interactions conducted in Petri dishes, multi-well plates, test tubes, and flow cytometry^20^. Importantly, we show that the applications of the assay could be extended to the study of neutrophil activity against other live pathogenic fungi clusters, including *C*. *auris*, *C*. *glabrata*, and *A*. *fumigatus*.

The process of neutrophil cooperation against large targets is also known as neutrophil swarming and has been observed using in vitro and in vivo models^7,21^. Recently, several of the mediators of these neutrophil–neutrophil interactions have been identified using inert microbe-like targets^6,8^. The findings of this study complement previous studies of neutrophil swarming and reveal several mechanisms that are important for microbe containment by neutrophil swarms. Moreover, through the use of inhibitors, animal models, and neutrophils from patients, new insight has emerged regarding the hierarchical order of importance for different antimicrobial mechanisms during fungal restriction.

The control of *C*. *albicans* during the early interactions between neutrophils and fungi appear to depend in part on MPO. Defects in MPO function result in control defects starting very early, during germination of hyphae. This antimicrobial role of MPO matches known functions in traditional studies in vitro and in animal models, including specific defects in control of *C. albicans*^22,23^. Interestingly, it has been suggested that MPO function becomes more important at higher microbial burdens, situations where neutrophil swarming could be more relevant^22–24^.

NADPH oxidase influences swarming dynamics and indirectly the containment of *C*. *albicans*. Interestingly, without ROS production, swarming was enhanced for neutrophils from CGD patients and for a mouse neutrophil line genetically deficient in NCF1. This role is consistent with known function for ROS in cell signaling, communication, and recruitment^25–30^. The anti-inflammatory role of ROS is also in line with data seen in CGD patients who can develop pathological, excessive inflammatory responses^31–33^. The increased swarming size is consistent with previous reports that CGD neutrophils release increased amounts of pro-inflammatory cytokine, interleukin (IL)-8, in response to fungi, including *C*. *albicans*, and increase accumulation of LTB_4_^34,35^. Both IL-8 and LTB_4_ are known key signals for human neutrophil swarming, as shown in recent studies using inert targets for swarming^6,8^. Despite these exaggerated swarming responses, NCF1 KO and CGD neutrophils were still deficient in *C*. *albicans* killing and restriction. These results suggest that a careful balance exists for ROS production where it is both destructive and antimicrobial while also influencing swarming dynamics to not become excessive. Interestingly, DPI and Apocynin, both frequently used as inhibitors of NADPH oxidase, induced swarming phenotypes different from the NCF1 KO and human CGD neutrophil. The differences may be explained by the off-target effects of DPI and Apocynin^36^. Apocynin has been published to also inhibit Rho kinases, and Rho kinases are also known to be involved in neutrophil chemotaxis^36–39^.

NET release plays a key role in the late phases of swarming interactions, restricting *C*. *albicans* that were not eliminated early on by the action of MPO, ROS, and other antimicrobial mechanisms. This role is consistent with recent in vivo studies showing that NETs localized around microbe clusters and biofilms restrict the ability of *Pseudomonas aeruginosa* in those biofilms to disseminate to the brain^40^. Interestingly, NET release occurred around every microbe cluster tested, in contrast with NET release against individual microbes that occurs only in a small subset of the interactions^41^. The level of NET release was larger against *C*. *albicans* germinating into hyphae compared to a yeast-locked strain. This is consistent with previous observations that NET production is influenced by microbial size^41^. Moreover, swarming and NET release were also larger against wild-type *C*. *albicans* than other Candida species that do not make hyphae, even when all were presented at the start as identically sized clusters, again suggesting a role for hyphae in inducing robust NET responses.

We found that the efficacy of neutrophil swarming against *C*. *albicans* can be enhanced by priming neutrophils with GM-CSF or GCSF. These factors appear to have significant impact early in the hierarchy of events during swarming, boosting neutrophil recruitment and direct fungal killing, and significantly extending the length of time it takes before surviving hyphae can escape the swarm. The contribution of GM-CSF or GCSF to enhancing the efficiency of neutrophil swarming against clusters of live microbes complements the known roles of these factors in stimulating neutrophil granulopoiesis^42–46^ and in boosting neutrophil antimicrobial actions, e.g., migration toward chemoattractants, production of ROS, phagocytosis, and the killing of bacteria and fungi^45,47,48^.

Consistent with these previous reports, we uncovered that GM-CSF or GCSF treatment can also enhance neutrophil function during MPO inhibition, significantly boosting neutrophil swarming recruitment. The fact that MPO deficiency can be easily overcome is in line with clinical data, as patients with partial or total MPO deficiency are not usually seen to be hypersusceptible to infections except those with underlying comorbidities, such as diabetes mellitus. It is likely that these patients could benefit from such cytokine treatments capable of boosting neutrophil and swarming functions^22,49,50^.

The potential of GM-CSF and GCSF to partially rescue neutrophil function during ROS deficiency or chemical inhibition is an exciting finding, as CGD patients are susceptible to frequent, life-threatening infections^28,51^. Intriguingly, GCSF and GM-CSF have been used as augmenting treatments in the care of CGD patients during infectious complications^52–54^. It is known that GM-CSF and GCSF do not restore the oxidative burst in CGD patient neutrophils, providing evidence that the impact seen in swarming is through a ROS-independent pathway^55^. Moving forward, systematic dissection of the mechanisms used by neutrophils to kill microbes and regulate their own function will be especially critical in CGD, where therapies to enhance microbial killing must be carefully balanced to not simultaneously exacerbate the inflammatory complications found in these patients^31–33^. A better understanding of swarming and its role(s) during infection and inflammation will ultimately expand the therapeutic options for patients at risk of infection or afflicted with inflammatory pathologies.

## Methods

### Study design

The objective of this study was to optimize an assay in which human neutrophil swarming against live microbes could be interrogated robustly and in molecular detail and to validate the assay using *C*. *albicans* as an example organism. To do this, we created poly-l-lysine/ZETAG microarrays that could hold clusters of live fungi and challenged them with human neutrophils. We monitored the progression of swarming and the growth (or restriction) of fungi by time-lapse microscopy. The accumulation of neutrophils (Hoechst staining), release of NETs (appearance of Sytox staining), and *C*. *albicans* viability were also monitored by fluorescence during time-lapse microscopy. Interrogation of individual molecular mechanisms was accomplished by using the appropriate inhibitor and matched vehicle controls in experiments. Isolated human neutrophils (same donor) were pooled and distributed randomly between conditions in each experiment, but otherwise no formal randomization or blinding was used. Sample size consisted of *N* = 3+ donors for all experiments unless noted otherwise. Sample size was *N* = <3 for experiments solely meant to confirm previously observed and published results (e.g., the role of LTB_4_ with inhibitors MK-886 and U75302) or those experiments not central to claims of the paper (e.g., swarming to *A. fumigatus*). For manual image analysis, a set of strict analysis rules were established prior to any processing to ensure equivalent and fair quantification in all conditions and experiments. The number of independent replicates for each experiment is outlined in the figure legends and summarized in Supplementary Table 1.

### Array printing

Utilizing a microarray printing platform (Picospotter, PolyPico, Galway, Ireland), we printed a solution of 0.1% poly-l-lysine (Sigma-Aldrich) and ZETAG targets with 100 µm diameter. For experiments, we printed 8 × 8 arrays in a 16-well format on ultra-clean glass slides (Fisher Scientific). Slides were screened for accuracy and then dried at 40 °C for 2 h on a heated block. After 2 h, slides were removed from the heat block and left at room temperature until required.

### Neutrophil isolation

Fresh samples of peripheral blood from healthy volunteers was collected in 10 mL heparin or EDTA tubes (Research Blood Components LLC, Allston, MA). Protocols were approved by the institutional review board at Massachusetts General Hospital (MGH). Blood was utilized within 6 h after the blood draw, except for experiments with CGD samples that had both CGD and control blood shipped overnight from the National Institutes of Health (NIH). Neutrophils were isolated using the EasySep Direct Human Neutrophil Isolation Kit per the manufacturer’s protocol (STEMCELL Technologies). Isolated neutrophils were stained with Hoechst (ThermoFisher Scientific) and re-suspended in Iscove’s Modified Dulbecco’s Media with 20% fetal bovine serum (ThermoFisher Scientific).

### CGD patients

Blood samples were obtained by phlebotomy from CGD patients, where the diagnosis was confirmed by molecular and/or functional testing. The collection of samples from consented patients was approved by the internal review board at the NIH under protocol # 93-I-0119 (NCT00001355) and institutional review board at the MGH. For matched controls, healthy volunteer blood was also collected at the same time and shipped under same conditions under the same approved protocols. Samples were all de-identified and shipped at room temperature in isothermal boxes from the NIH to the MGH. The patient and control blood samples were received and processed immediately upon receipt the following morning.

### Microorganism culture

*C*. *albicans*, yeast-locked *C*. *albicans*, *C*. *auris*, and *C*. *glabrata* were inoculated into fresh yeast extract peptone dextrose liquid media and grown shaking at 30 °C overnight. The far red fluorescent and yeast-locked *C*. *albicans* was a kind gift from Robert Wheeler (University of Maine)^56,57^. Wild-type *C*. *glabrata* (ATCC2001) was purchased from the American Type Culture Collection (ATCC, Manassas, VA). A clinical isolate of *C*. *auris* was obtained from the MGH microbiology laboratory (Boston, MA). *A*. *fumigatus* conidia of strain 293 expressing cytosolic red fluorescent protein was a kind gift from Jatin Vyas (MGH), prepared by Nida Khan using standard methods. Upon receipt, conidia were filtered using spin columns with 5-μm pores (Thermo Scientific) to isolate un-germinated spores and stored in dH_2_O at 4 °C until required.

### Target patterning

Sixteen-well ProPlate wells (Grace Bio-labs) were attached to glass slides with printed arrays. Fifty µL of a suspension of the desired target, either live microorganisms grown as outlined above or fluorescent microbe-derived bioparticles (ThermoFisher Scientific), was added to each well and incubated with rocking for 5–10 min. Following incubation, wells were thoroughly washed out with phosphate-buffered saline (PBS) to remove unbound targets from the glass surface. Wells were screened to ensure appropriate patterning of targets onto the spots with minimal non-specific binding before use.

### Swarming experiments

All imaging experiments were conducted using a fully automated Nikon TiE microscope. Time-lapse imaging was conducted using a ×10 Plan Fluor Ph1 DLL (NA = 0.3) lens and endpoint images were taken with a ×2 Plan Apo (NA = 0.10) lens. Confocal imaging was conducted using a ×40 Plan Fluor (NA = 0.75) lens. Swarming targets to be observed during time lapse were selected and saved using the multipoint function in NIS elements prior to loading of neutrophils. Five hundred thousand neutrophils were added to each well unless otherwise noted. All selected points were optimized using the Nikon Perfect Focus system before launching the experiment. In experiments using chemical inhibitors, neutrophils were pre-incubated with the chemical or appropriately matched vehicle control for 30 min before use.

### NETosis visualization and chemical inhibitors

To visualize NET formation, Sytox green was added to the media at 500 nM (ThermoFisher Scientific). For inhibition of MPO activity, we included ABAH in the media at a concentration of 50 µM (Cayman Chemicals). For inhibiting the phagocyte oxidase and ROS generation, we included DPI (Sigma-Aldrich) in the media at a concentration of 10 µM, Apocynin (Cayman Chemicals) at a concentration of 300 µM, or Trolox (Cayman Chemicals) at a concentration of 500 µM. To disrupt LTB_4_ synthesis, MK-886 was included at 1 µM (Cayman Chemicals). BTL1 receptor blocking was done with U75302 at a concentration of 1.38 µM (Cayman Chemicals). All chemical inhibitors were accompanied and compared to the appropriate vehicle control (dimethyl sulfoxide for ABAH, DPI, Apocynin, Trolox, and MK-886, ethanol for U75302). GM-CSF was used at a concentration of 0.2 ng/mL, and GCSF was used at a concentration of 300 ng/mL. To visualize citrullinated histone H3 in NETs without fixation, we modified an existing live NET staining protocol^58^. Briefly, we set up the assay as described above and allowed swarming to proceed for 5 h. At this time, media was removed from the wells and replaced with the blocking solution. Samples were then stained with Anti-Histone H3 citrulline R2 + R8 + R17 (abcam; 0.014 mg/mL) followed by donkey anti-rabbit IgG Cy3 (Jackson Immunoresearch; 0.0075 mg/mL) secondary antibody. The array wells were then removed and replaced with a coverslip before confocal imaging.

### Image analysis

Area analysis was performed manually by outlining the swarms or areas of fungal growth in the NIS-elements (v4.00.12; Nikon Inc.) or FIJI (FIJI is just ImageJ v2.0.0-rc-59/1.52p, NIH) software. For area of the swarm, only the swarm itself (just the neutrophils) was measured. This was done using the 4,6-diamidino-2-phenylindole fluorescent channel image, using Hoechst staining to identify neutrophils. For areas of fungal growth, a combination of brightfield and fluorescent channels were used. Fungi used in experiments were always fluorescent, except for *C*. *auris*. We combined the appropriate fluorescent channel with the brightfield image to be sure we included any escaped fungal elements, like lone hyphae, that may not show up well in the fluorescent channel. For scoring of germination, we observed the fluorescent fungi in each swarm, determining the frame when germination becomes visible. For scoring of hyphal escape, we observed the fluorescent fungi in each swarm via time-lapse and determined the time when hyphae first breach the containment of the swarm (extends beyond the area of neutrophils). For escape in the case of *C*. *albicans* with no neutrophils, escape was instead defined as escape from an area equivalent to the area of an average swarm. Intensity profiles were generated by defining regions of interest and using the time measurement option in the NIS-elements or FIJI software.

### Neutrophil cell lines

For construction of the NCF1-deficient cells, we used a granulocyte-monocyte progenitor (GMP) cell line that is conditionally immortalized by the expression of an estrogen receptor-homeobox B8 (ER-HoxB8) gene fusion, which are maintained in the M.K.M. laboratory and have been described previously^59^. Briefly, media contains stem cell factor and estradiol, which permits nuclear translocation of the ER-HoxB8 fusion protein resulting in a conditional maturation arrest at the GMP stage. Removal of estradiol allows synchronous differentiation of the GMP into mature neutrophils^60^. All GMP cell lines were cultured in complete RPMI media containing 2% stem cell factor-conditioned media and 0.5 μM β-estradiol and grown in a humidified incubator at 37 °C in the presence of 5% CO_2_ until ready for use. GMPs were removed from estrogen-containing media and matured into functional neutrophils for 4 days prior to experiment use. When ready, control or NCF1-deficient neutrophils derived from these lines were treated exactly as described above for human neutrophils when running swarming assays.

### CRISPR-Cas9 system and single-guide RNA (sgRNA) design

The lentiCRISPR vector was purchased from Genetic Perturbation Platform, Broad Institute (Cambridge, MA)/Addgene (Cambridge, MA). To construct the lentiviral sgRNA Cas9 vector, sgRNAs were cloned into lentiCRISPR vector in the BsmBI site^60^. Lentivirus production and purification were performed as previously described^61^. sgRNAs were designed using the CRISPR tool^62^ (http://crispr.mit.edu) to minimize potential off-target effects. The lentiCRISPR with sgRNAs targeting *NCF1* were cloned using the following sequences: *NCF1* sgRNA1: GCCCCTTGACAGTCCCGACG (A7 G5-1) and *NCF1* sgRNA2: CGTTGCCCATCAAACCACCT (A9 A5-1). Lentiviral infection was carried out in a fibronectin (Millipore, Burlington, MA) coated 12-well plate. In all, 5 × 10^5^ cells were infected with *NCF1* sgRNA containing lentivirus by spinoculation (970 × *g*, 60 min, 25 °C), in the presence of 24 µg/mL polybrene (Millipore, Burlington, MA).

### Western blot

Neutrophils were lysed in reducing agent (NuPAGE® Sample Reducing Agent, ThermoFisher) and sodium dodecyl sulfate (SDS) sample buffer (4× Laemmli Sample Buffer, BioRad). Proteins were resolved by SDS-polyacrylamide gel electrophoresis under reduced conditions and transferred onto a polyvinylidene difluoride membrane. Membranes were blocked in PBS–1% Tween with 5% nonfat milk. Total NCF1 protein was detected with p47 phox (NCF1) D-10 (1:250; Santa Cruz Biotechnology, Dallas, TX). The blots were subsequently reacted with mouse monoclonal antibody AC-15 anti–β-actin (1:200,000; Sigma, St. Louis, MO).

### ROS production

ROS production was measured as described^63^. Briefly, neutrophils were plated at 5 × 10^5^ cells per well in cRPMI, in 96-well white-wall plates (Grenier Bio-One, Monroe, NC). Cells were placed on ice and heat-killed *C*. *albicans* hyphae were added at a multiplicity of infection of 10. A lucigenin solution was added to each well for a final concentration of 15 µM lucigenin in cRPMI. Luminescence was measured at 37 °C every 5 min for 4 h in a SpectraMax i3x reader (Molecular Devices, San Jose, CA) and expressed as arbitrary fluorescence units.

### Statistics and reproducibility

Data were tested for normality using a D’Agostino–Pearson omnibus normality test. Normally distributed data were analyzed with Student’s *T* test or one-way analysis of variance with Tukey’s post-test. Non-normally distributed data were analyzed with Mann–Whitney or Kruskal–Wallis with Dunn’s post-test where appropriate. Statistical significance was considered for *p* < 0.05; exact *p* values are provided in the relevant figure legends. All statistics were conducted using the GraphPad Prism 7.03 software. For figures with micrographs (Figs. 1a; 2a; and 4c, d; Supplementary Figs. 1a; 3a; 4; 5a; 6a, b; 7a, b; 8b; 9a), these images are representative of three independent runs, with the following exceptions: Figure 1a represents two donors. Figure 2a is representative of healthy control neutrophils, which have been run for over 40 independent times in this assay. Figure 4c, d is representative of one healthy control run and three unique CGD samples. Supplementary Fig. 1a shows representative fungal growth, which has been run >40 independent times for *C*. *albicans* and >10 independent times for *C*. *glabrata*, *C*. *auris*, and *A*. *fumigatus*. Supplementary Fig. 5a is representative of two independent runs. Supplementary Fig. 9a is representative of four independent runs. Full sample information for all data tested (discrete *N*, number of donors, etc.) are included in Supplementary Table 1.

### Reporting summary

Further information on research design is available in the Nature Research Reporting Summary linked to this article.

## Supplementary information

Supplementary Information

Description of Additional Supplementary Files

Supplementary Movie 1

Supplementary Movie 2

Reporting Summary

## Data Availability

The source data for Figs. 1b–e; 2b–e; 3b–f; 4a, b, e–h; 5a–e; 6a–e; and 7a–h and Supplementary Figs. 2, 3b–f; 5b, c; 6c–e; 7c–f; 8a, b; 9b–f; 10a–e; and 11a–l are provided as a Source Data file. All other data are available from the authors upon request.

## Source Data

Source Data
