# Publisher Correction: Neutrophil swarming delays the growth of clusters of pathogenic fungi

**DOI:** 10.1038/s41467-020-16446-8

**Published:** 2020-05-14

**Authors:** Alex Hopke, Allison Scherer, Samantha Kreuzburg, Michael S. Abers, Christa S. Zerbe, Mary C. Dinauer, Michael K. Mansour, Daniel Irimia

**Affiliations:** 1 BioMEMS Resource Center, Massachusetts General Hospital, Boston, MA 02129 USA; 2 Harvard Medical School, Boston, MA 02115 USA; 3 Shriners Hospital for Children, Boston, MA 02114 USA; 4 Division of Infectious Diseases, Massachusetts General Hospital, Boston, MA 02114 USA; 5 Laboratory of Clinical Immunology and Microbiology (LCIM), National Institute of Allergy and Infectious Diseases (NIAID), National Institutes of Health (NIH), Bethesda, MD 20814 USA; 6 Departments of Pediatrics and of Pathology and Immunology, Washington University School of Medicine in St. Louis, St. Louis, MO 63110 USA

**Keywords:** Infectious diseases, Innate immune cells, Fungal host response

Correction to: *Nature Communications* 10.1038/s41467-020-15834-4, published online 27 April 2020.

The original version of this Article contained an error in the spelling of the author Alex Hopke, which was incorrectly given as Hopke Alex.

This has now been corrected in both the PDF and HTML versions of the Article.

