# Supplementary material for: Neutrophil swarming delays the growth of clusters of pathogenic fungi
Source: Nat Commun. 2020 Apr 27;11:2031. doi: 10.1038/s41467-020-15834-4 (PMC7184738; doi:10.1038/s41467-020-15834-4)
Supplement: Supplementary file 1 — Supplementary Information [file 41467_2020_15834_MOESM1_ESM.pdf]

Supplementary Information:

Neutrophil swarming delays the growth of clusters of pathogenic fungi

Hopke et al.

Supplementary Table 1: Summary of Swarming Experiments

The number of individual swarms quantified along with meaning of the error bars in each timepoint and the condition and number of donors involved in each experiment are shown. Overall, more than 7,000 swarms were observed over time across all experiments. Standard deviation, SD. Standard error, SE.

Panel	Individual Points	Error bars	Number of Donors
Figure 1b	Average of 24 swarms for 100+, 42 for 100-20 and 29 for the 20-0 groups.	SD	2
Figure 1c	21 swarms for the transient, 7 for the delayed and 15 for the dynamic group.	none (individual tracks shown)	2
Figure 1d MK-886	144 swarms	SD	3
Figure 1d U75302	96 swarms from a representative donor	SD	3
Figure 1d PMN	240 swarms	SD	3
Figure 1e	192 swarms per group	SD	3
Figure 2b	40 swarms	SD	3
Figure 2c	40 swarms	SD	3
Figure 2d	104 swarms for all points except 6 and 8 hours, which have 72	SD	3
Figure 2e *C. albicans*	182 swarms	SD	3
Figure 2e *C. albicans* yeast locked	192 swarms	SD	3
Figure 2e *C. auris*	73 swarms	SD	3
Figure 2e *C. glabrata*	199 swarms	SD	3
Figure 2e *A. fumigatus*	48 swarms	SD	1
Figure 3b	68 swarms except the 12 hour point which has 60 swarms	SD	3
Figure 3c	44 swarms	SD	3
Figure 3d	12 swarms	SD	3
Figure 3e	12 swarms	SD	3
Figure 3f	144 swarms except the PMN control group which has 95 swarms	SD	3
Figure 4a DPI	36 swarms	SD	3
Figure 4a Apocynin	48 swarms	SD	3
Figure 4d	MFI from a single representative field of view	none (individual track shown)	3
Figure 4b PMN	288 swarms	SD	3
Figure 4b PMN +Trolox	96 swarms	SD	3
Figure 4b PMN + Apocynin	286 swarms	SD	3
Figure 4b No PMN	219 swarms	SD	3
Figure 4e Control	16 swarms	SD	1
Figure 4e CGD	48 swarms	SD	3
Figure 4f Control	96 swarms	SD	1
Figure 4f CGD	288 swarms	SD	3
Figure 4g	16 swarms	SD	1
Figure 4h	16 swarms	SD	1
Figure 5a	40 swarms	SD	3
Figure 5b	40 swarms	SD	3
Figure 5c	240 swarms	SD	3
Figure 5d	16 swarms from a representative donor	SE	4
Figure 5e	16 swarms from a representative donor	SE	4
Figure 6a	357 swarms for Control, 331 for GCSF and 309 for GM-CSF	SD	4
Figure 6b	40 swarms	SD	3
Figure 6c	40 swarms	SD	3

Figure 6d	285 swarms for Control, 271 for GCSF and 284 for GM-CSF	SD	4
Figure 6e	16 swarms from a representative donor	SE	4
Figure 7a	40 swarms	SD	3
Figure 7b	40 swarms	SD	3
Figure 7c	256 swarms PMN, 226 Apocynin, 210 GCSF +Apocynin, 208 GM-CSF + Apocynin, 233 Candida Alone	SD	3
Figure 7d	16 swarms from a representative donor	SE	4
Figure 7e	40 swarms	SD	3
Figure 7f	40 swarms	SD	3
Figure 7g	341 swarms PMN, 355 ABAH, 342 GCSF +ABAH, 349 GM-CSF + ABAH, 323 Candida Alone	SD	4
Figure 7h	16 swarms from a representative donor, except for GM-CSF and GCSF which represent 15 swarms	SE	4
Fig S2	24 swarms for 100+, 42 for 100-20 and 29 for the 20-0 group.	none (individual tracks shown)	2
Fig S3b	8 swarms from a representative donor	SE	3
Fig S3c	8 swarms from a representative donor	SE	3
Fig S3d	8 swarms from a representative donor	SE	3
Fig S3e	45 swarms	SD	3
Fig S3f	32 swarms for the pmn points, 16 swarms for the C. auris alone points	SD	2
Fig S5b	8 swarms from a representative donor	SE	2
Fig S5c	16 swarms	SD	2
Fig S6c	68 swarms for *S. aureus* points and 60 swarms for *E. coli* points.	SD	3
Fig S6d	12 swarms for both *S. aureus* and *E. coli* points	SD	3
Fig S6e	12 swarms for both *S. aureus* and *E. coli* points	SD	3
Fig S7c	40 swarms	SD	3
Fig S7d	40 swarms	SD	3
Fig S7e	MFI from a single representative field of view	none (individual track shown)	3
Fig S7f Candida	88 swarms	SD	3
Fig S7f Candida +DPI	91 swarms	SD	3
Fig S7f Candida +Apocynin	88 swarms	SD	3
Fig S7f Candida +Trolox	83 swarms	SD	3
Fig S8a	9 wells	SE	3
Fig S9b	24 swarms	SD	3
Fig S9c	64 swarms	SD	4
Fig S9d	64 swarms except for GCSF, which represent 48 swarms	SD	4
Fig S9e	64 swarms except A5 GMCSF, A5 GCSF and G5 GCSF which represent 48 swarms	SD	4
Fig S9f	64 swarms except for NFC1 G5 and A5 treated with apocynin, which represent 48 swarms	SD	4
Fig S10a	16 swarms	SD	1
Fig S10b	16 swarms	SD	1
Fig S10c	16 swarms	SD	1
Fig S10d Control	16 swarms	SE	1
Fig S10d CGD	48 swarms	SD	2
Fig S10e Control	16 swarms	SD	1
Fig S10e CGD	48 swarms	SD	3
Fig S11a	16 swarms from a representative donor, except for GCSF which represents 15 swarms	SE	4
Fig S11b	16 swarms from a representative donor	SD	4
Fig S11c	16 swarms from a representative donor	SE	4

Fig S11d	16 swarms from a representative donor	SD	4
Fig S11e	16 swarms from a representative donor	SE	4
Fig S11f	16 swarms from a representative donor	SD	4
Fig S11g	16 swarms from a representative donor	SE	4
Fig S11h	16 swarms from a representative donor	SD	4
Fig S11i	16 swarms from a representative donor for Control, 12 for ABAH, 15 for GM-CSF and 14 for GCSF	SE	4
Fig S11j	16 swarms from a representative donor	SD	4
Fig S11k	16 swarms from a representative donor except for GM-CSF which represents 15 swarms	SE	4
Fig S11l	16 swarms from a representative donor	SD	4

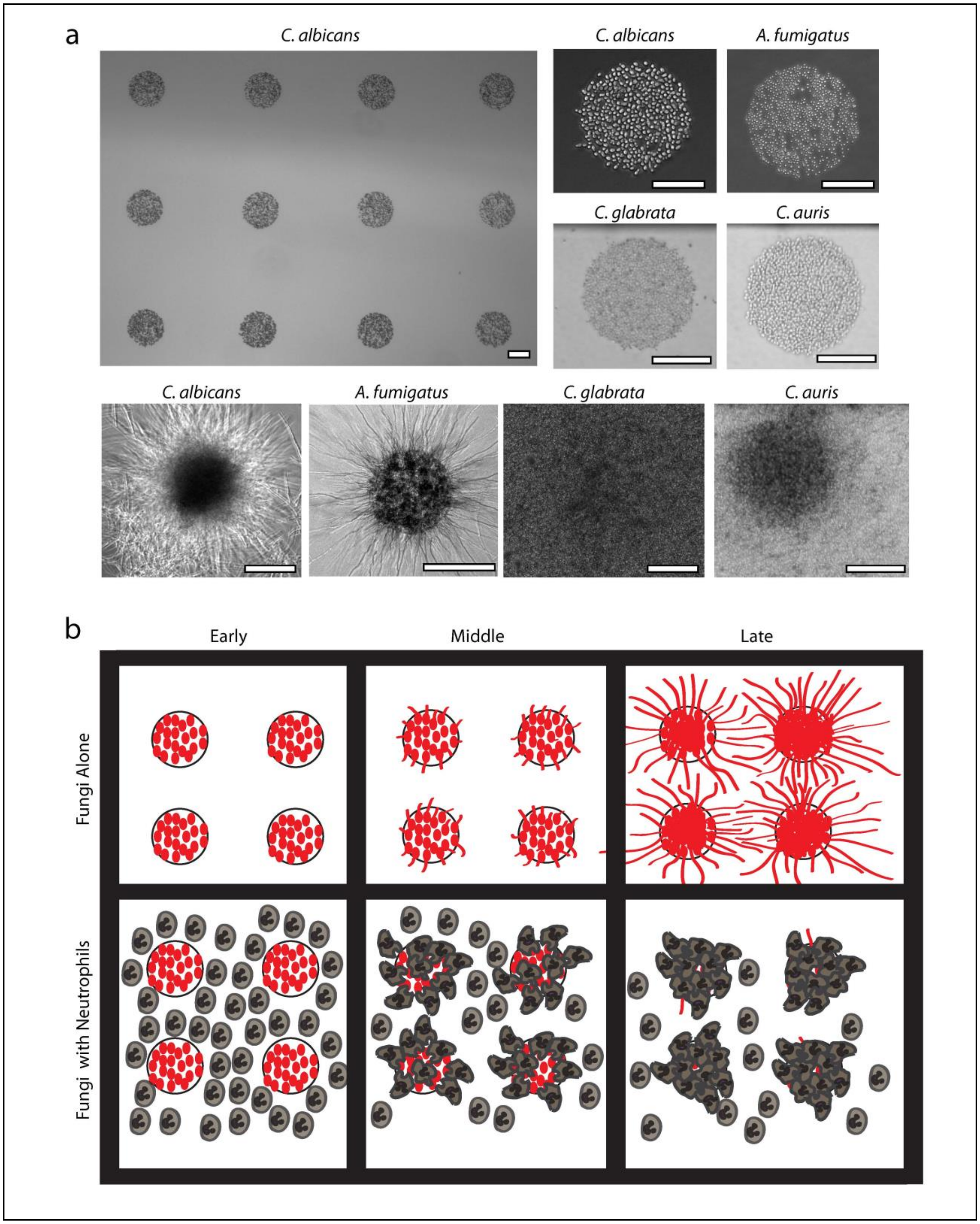

Supplementary Figure 1: Patterning of Live Fungi.

Living fungi were patterned in clusters on poly-l-lysine/zetag arrays. Fungi successfully patterned included the yeast of *C. albicans, C. glabrata* and *C. auris* as well as the conidia of *A. fumigatus* (a). Fungi incubated on our arrays grew at rates comparable to traditional culture. *C. albicans* and *A. fumigatus* forming hyphae that radiated out from the spots and *C. glabrata* and *C. auris* covering the slides in yeast. A cartoon model of the assay is shown in (b). The top row depicts the germination and growth of *C. albicans* when incubated on the arrays alone, while the bottom rows depict the restriction of this hyphal growth during human neutrophil swarming. Scale bars represent 100 µm for upper left panel and lower row of images, 50 µm for upper right set of four images.

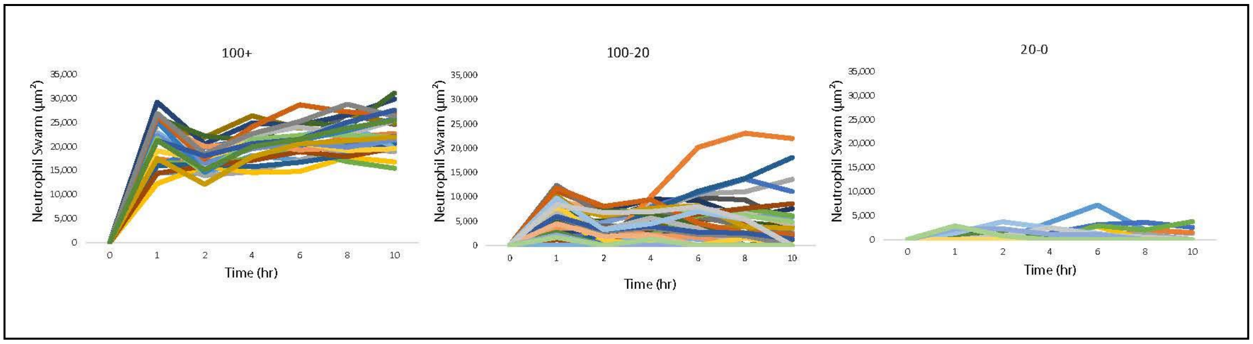

Supplementary Figure 2: Dynamics of Human Neutrophil Swarming to *C. albicans*.

Individual swarm tracks are shown for the different *C. albicans* density groups, highlighting the impact of *C. albicans* density on swarm dynamics. *C. albicans* density is indicated above the corresponding panels.

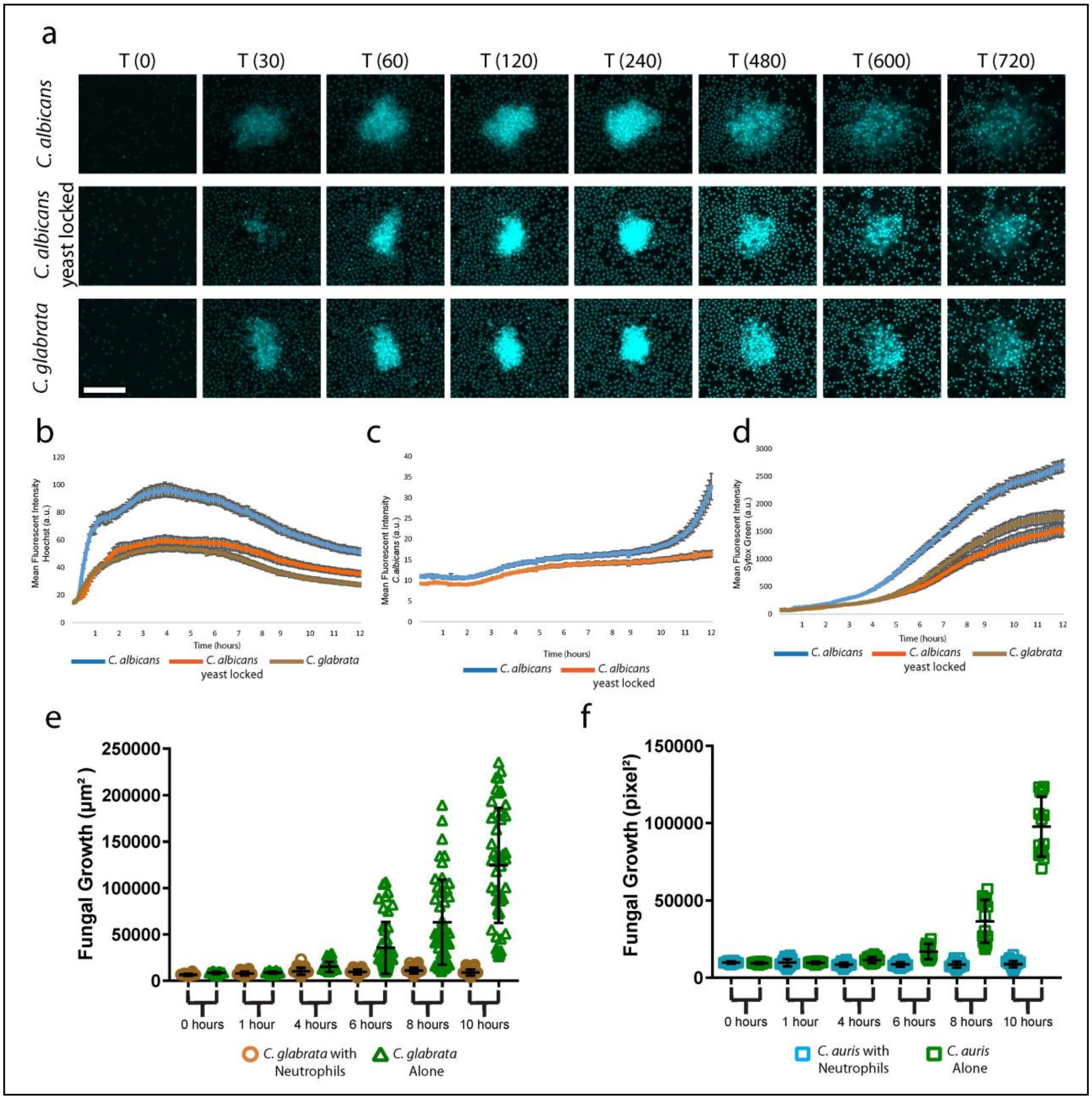

Supplementary Figure 3: Yeast-locked Candida Strains and Species Elicit a Reduced Swarming Response

Timelapse imaging shows the progression of neutrophil swarming against wild type *C. albicans*, yeast-locked *C. albicans* or *C. glabrata* are shown (a). The DAPI channel is presented. Wild type *C. albicans* induces a more robust swarming response. The MFI of Hoechst was quantified and is shown. Average of 8 swarms from a representative donor is shown (b). Yeast locked *C. albicans* is controlled more

effectively than wild type *C. albicans*. The MFI of *C. albicans* was quantified and is shown. Average of 8 swarms from a representative donor is shown (c). Wild type *C. albicans* induces a more robust NETosis response. The MFI of Sytox Green was quantified and is shown. Average of 8 swarms from a representative donor is shown (d). Quantification of the area of fungal growth during incubation of the *C. glabrata* or *C. auris* with or without neutrophils over time, showing that human neutrophil swarming restricts the growth of these fungi (d-e). N= 45 swarms across three different donors for *C. glabrata* (e). N=32 swarms for the "with neutrophils" group and N=16 spots for the *C. auris* alone group, across experiments with two different donors (f). Error bars represent mean +/- standard error for b-d and mean +/- standard deviation for e-f. Scale bar is 100 µm.

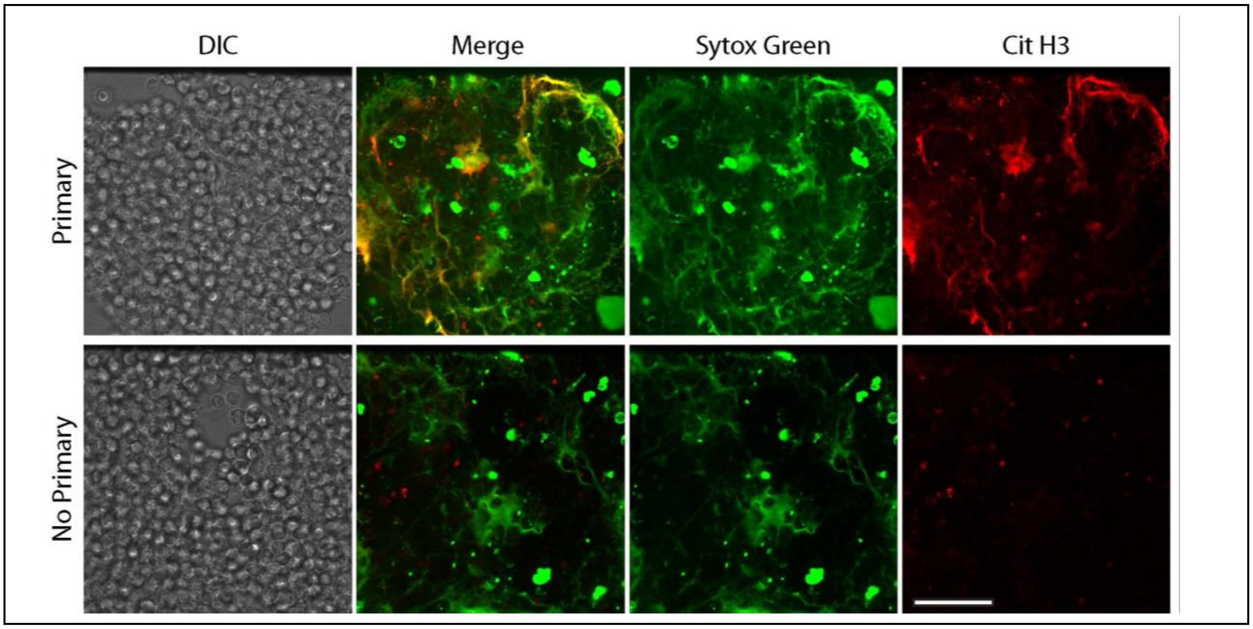

Supplementary Figure 4: Staining of NETs in Human Swarms

Human neutrophils were allowed to swarm against *C. albicans* for 5 hours. Swarms were then stained, without fixation, to show DNA (Sytox Green) and citrullinated histone H3. Swarms stained with secondary antibody but no primary antibody served as a control. Images represent maximum image projections for fluorescent channels and a single representative slice for the DIC. Scale bar 50 μm.

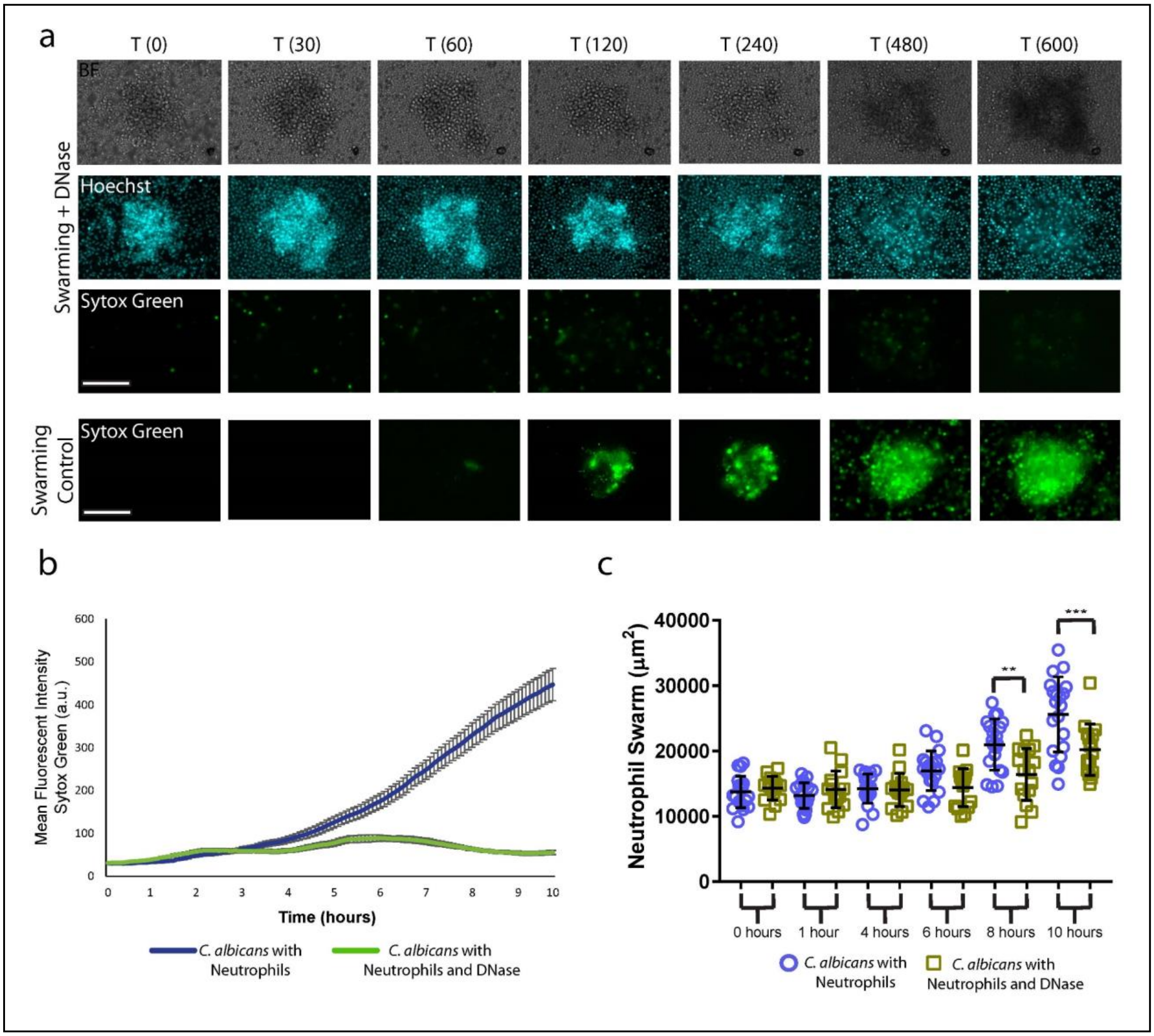

Supplementary Figure 5: DNase Effectively Degrades NETs During Swarming and Allows Candida Growth

Timelapse imaging shows the progression of swarming in the presence of DNase (200U). BF, DAPI (Hoechst) and FITC (Sytox Green) channels are shown (a). The FITC channel is shown for a swarm without DNase treatment as a comparison (fourth row). DNase degrades NETs. The MFI of Sytox Green staining was quantified for swarms with and without 200U of DNAase treatment. Average of 8 swarms from a representative donor is shown (b). NETs contribute to the expansion of the swarm at later timepoints. The area of the swarm was quantified over time for swarms with and without DNase

treatment. N= 16 swarms across two donors (c). ** $p= 0.0077$ and ***$p= 0.0004$ One way ANOVA with Tukey's post-test. Error bars represent mean +/- standard error for (b) and mean +/- standard deviation for (c). Scale bar represents 100 µm.

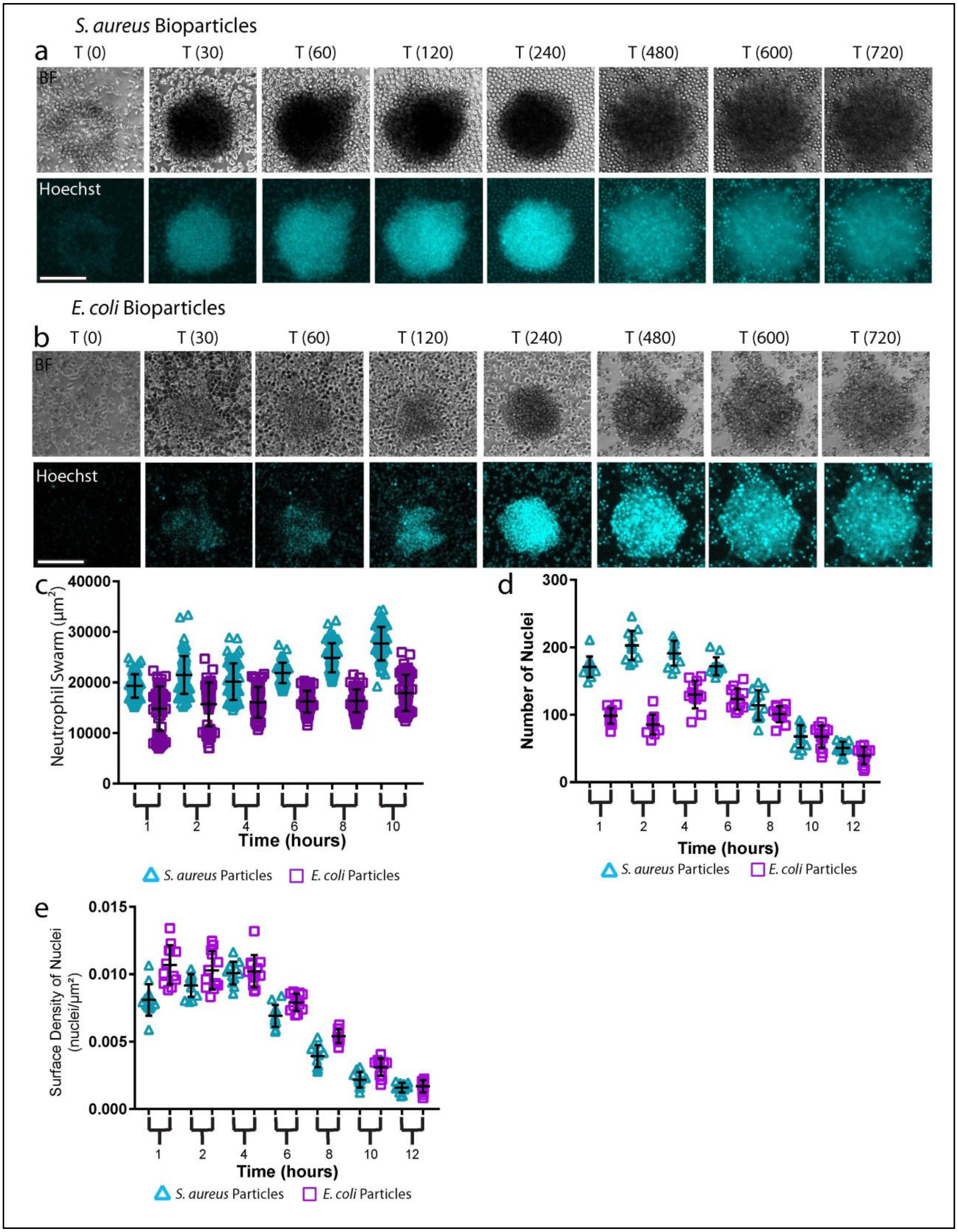

Supplementary Figure 6: Human Neutrophils Release NETs during Swarming against Multiple Microbial Targets

Human neutrophils were incubated with microbial particle arrays, including those derived from *S. aureus* and *E. coli*. Representative panels from a time-lapse showing the progression of neutrophil swarming to *S. aureus* or *E. coli* are shown, including the bright-field and DAPI (Hoechst staining) channels (a-b). The area of neutrophil swarms around *S. aureus* and *E. coli* particles (c), along with the number of nuclei (d) and the number of nuclei/μm^2 (e) was also quantified. N=68 swarms for *S. aureus* and N=60 swarms for *E. coli* across three donors for the areas (c). N=12 swarms for d and e. Error bars represent mean +/- standard deviation. Scale bar represents 100 µm.

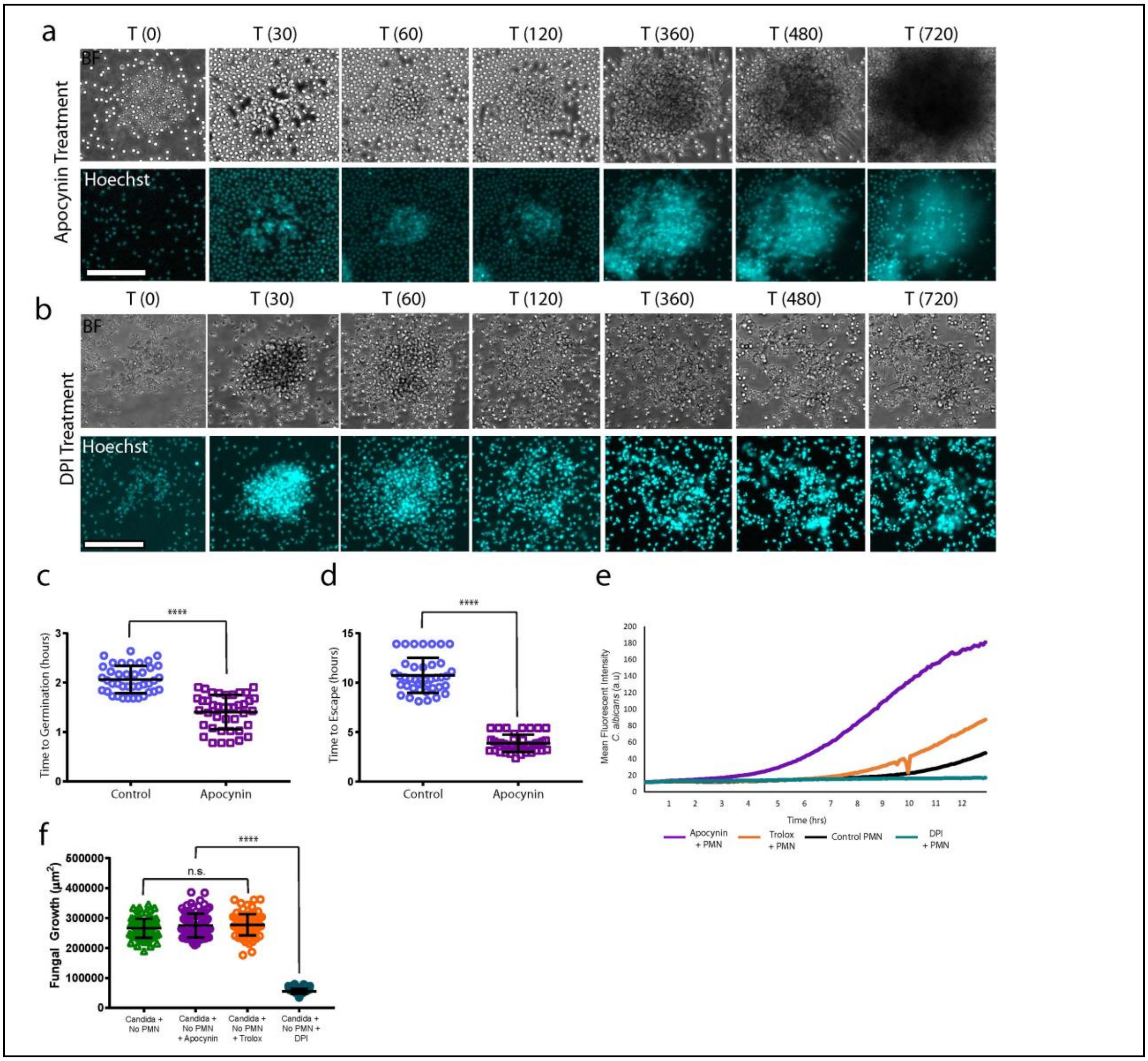

Supplementary Figure 7: ROS Inhibition by Apocynin and DPI Results in Opposing Phenotypes

Human neutrophils were incubated with ROS inhibitors to interrogate the mechanisms necessary to restrict the growth of *C. albicans* during swarming. Human neutrophil swarming is regulated by reactive oxygen species. Apocynin (300 µM) disrupted the ability of neutrophils to swarm to *C. albicans*, while the inclusion of DPI (10 µM) resulted in rapid formation and then dissolution of swarms, as shown in a representative series of image panels (a-b). *C.albicans* within swarms treated with Apocynin germinate faster than in vehicle treated control swarms. N= 40 swarms across 3 donors (c). Fungal hyphae penetrated and escaped containment by neutrophil swarms faster during Apocynin treatment. N= 40

swarms across 3 donors (d). The inhibition of ROS production significantly disrupted the ability of swarming to restrict *C. albicans* growth. The growth of *C. albicans* during swarming, as measured by expression of a far-red fluorescent protein, is shown in the presence of different ROS inhibitors (e). The area of fungal growth when *C. albicans* is incubated with the ROS inhibitors without neutrophils is also shown, demonstrating that DPI has a direct impact on *C. albicans* (f). N=88 spots for *C. albicans* only and *C. albicans* + APocynin, N=83 spots for *C. albicans* + Trolox and N=91 spots for *C. albicans* +DPI. ****p <0.0001 Students T-test (unpaired, two-tailed) for c,d and Kruskal-Wallis with Dunns multiple comparison test for f. Error bars represent mean +/- standard deviation. Scale bar is 100 µm.

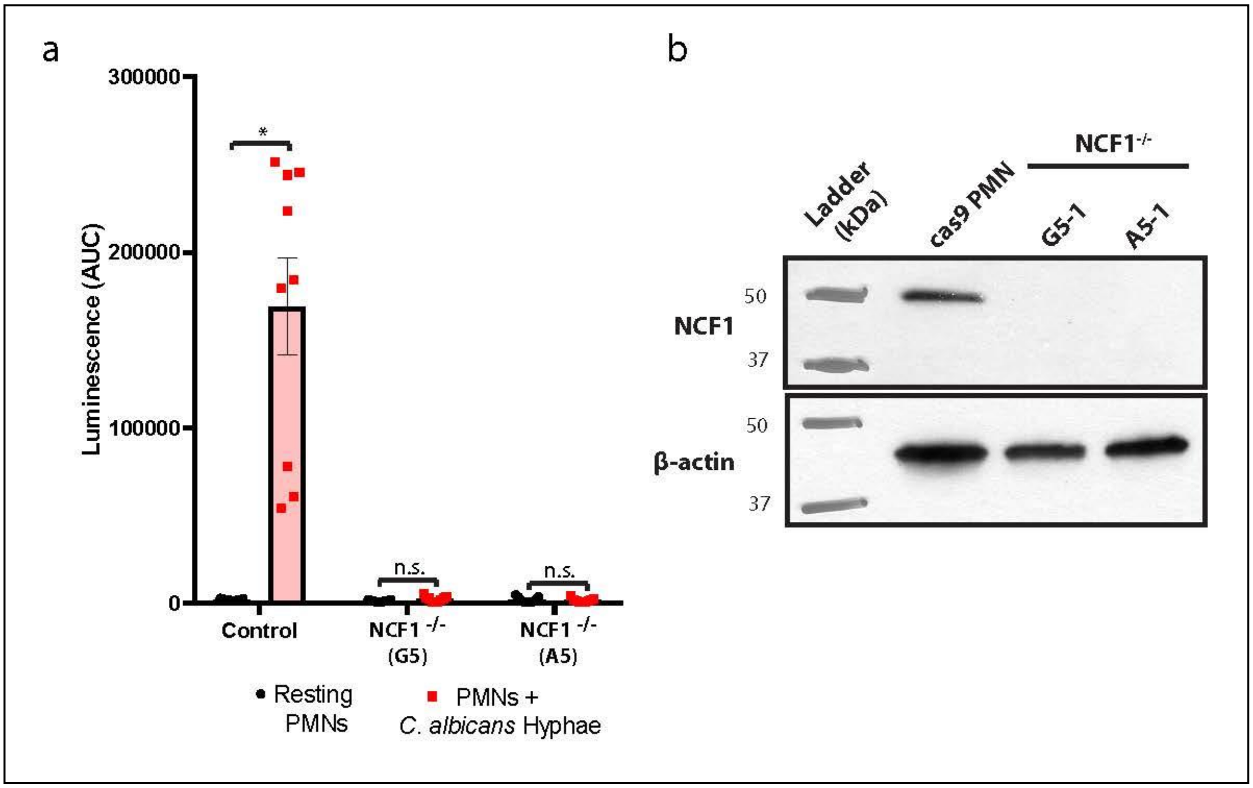

Supplementary Figure 8: Validation of NCF1 KO Cells

NCF1 KO clones were validated by functional assays and by Western Blot. NCF1 deficient clones are severely defective in ROS production during incubation with *C. albicans* heat-killed hyphae. Total lucigenin luminescence corresponds with ROS production (AU, arbitrary units). N=9 wells pooled from 3 independent runs (a). Western blotting of NCF1 protein comparing parental Cas9 control neutrophils and NCF1 knockout ER-HoxB8 neutrophils generated using CRISPR-Cas9. Western blot confirmed the loss of NCF1 protein in the mature neutrophil cell lines (b). β-actin serves as a standard protein loading control. *p = 0.0106, n.s. is non-significant Kruskal-Wallis with Dunns post-test. Error bars represent mean +/- SEM.

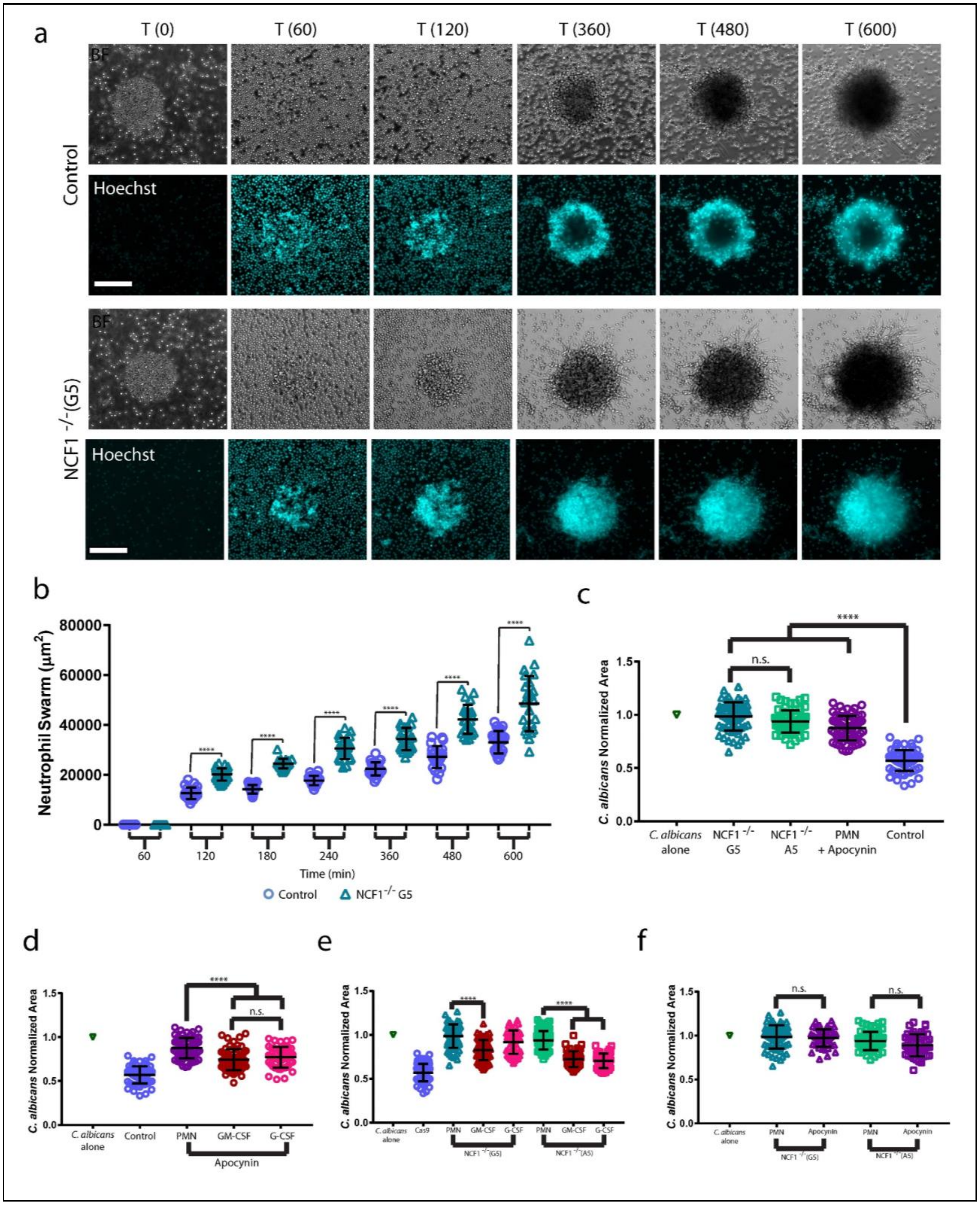

Supplementary Figure 9: NCF1 deficiency Results in Boosted Neutrophil Swarming but Reduced Fungal Control

Control or NCF1 deficient mouse neutrophils matured from the Hoxb8 cell system were added to swarming arrays of *C. albicans*, exactly as described for human cells. Representative panels are shown from a timelapse showing the progression of swarming by control or NCF1 deficient neutrophils, including brightfield and DAPI (Hoechst staining) channels (a). The area of the neutrophil swarm was quantified at specified time points duing the assay (b). N=24 swarms across three independent runs. The area of fungal growth was quantified at 10.5 hours into the assay and normalized to the growth of *C. albicans* alone (c-f). ROS inhibition by apocynin or NCF1 deficiency results in significantly more fungal growth than during control swarming. N= 64 swarms across 4 independent runs (c). Treatment of mouse HoxB8 control cells with GM-CSF or GCSF can partially rescue antifungal action during apocynin treatment. N= 64 swarms except for the Apocynin GCSF group for which N=48 swarms across 4 independent runs (d). Treatment of NCF1 deficient neutrophils can partially rescue antifungal action. N= 64 swarms except for G5 GCSF, A5 GM-CSF and A5 GCSF for which N=48 across 4 independent runs (e). Treatment of NCF1 deficient cells with apocynin has no significant impact on antifungal control. N=64 swarms except for G5 and A5 Apocynin groups for which N=48 swarms across 4 independent runs (f). ****$p<0.0001$ One way ANOVA with Tukeys post-test for b-d and f or Kruskal-Wallis with Dunns post-test for e. Error bars represent mean +/- standard deviation. Scale bar represents 100 μm.

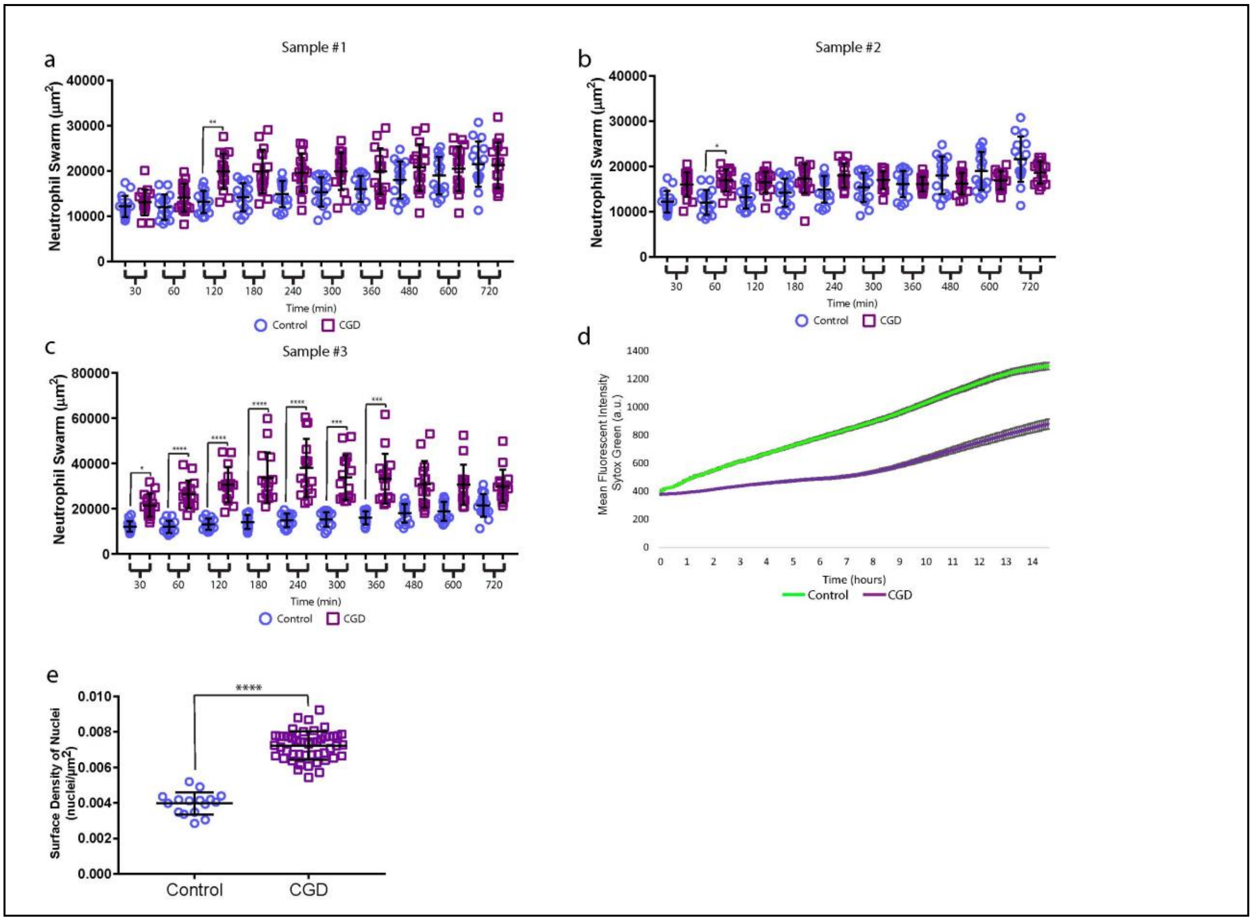

Supplementary Figure 10: Individual CGD Sample Profiles

CGD neutrophils exhibit increased swarming at early timepoints, though the extent is variable. The area of neutrophil swarms were quantified and shown for each CGD sample individually (a-c, the pooled results can be seen in **Fig 4e**). N=16 swarms for healthy donor and for each CGD sample except for CGD timepoints 240-720 for sample#2 and 120-720 for sample#3, for which N=15 swarms. CGD samples show reduced NET release. The MFI of Sytox Green staining was quantified for swarms from a healthy donor and from CGD neutrophils (d). The average is shown for 16 swarms from a healthy donor and 48 swarms across three CGD samples. The number of nuclei/µm^2 was quantified and is shown (e). N=16 swarms for healthy donor and 48 swarms across three CGD samples. *$p \leq 0.05$ ($p=0.0267$ for a, $p=0.0388$ for c) **$p \leq 0.01$ ($p=0073$ for a), ***$p \leq 0.001$ ($p= 0.0001$ for control 300 vs CGD 300 and $p=0.0007$ for

control 360 vs CGD 360 in c), ****p <0.0001 (c, e). Kuskal-Wallis with Dunn's post-test a-c; Mann-Whitney for f (unpaired, two-tailed). Error bars represent mean +/- standard deviation except for d, which represents mean +/- standard error.

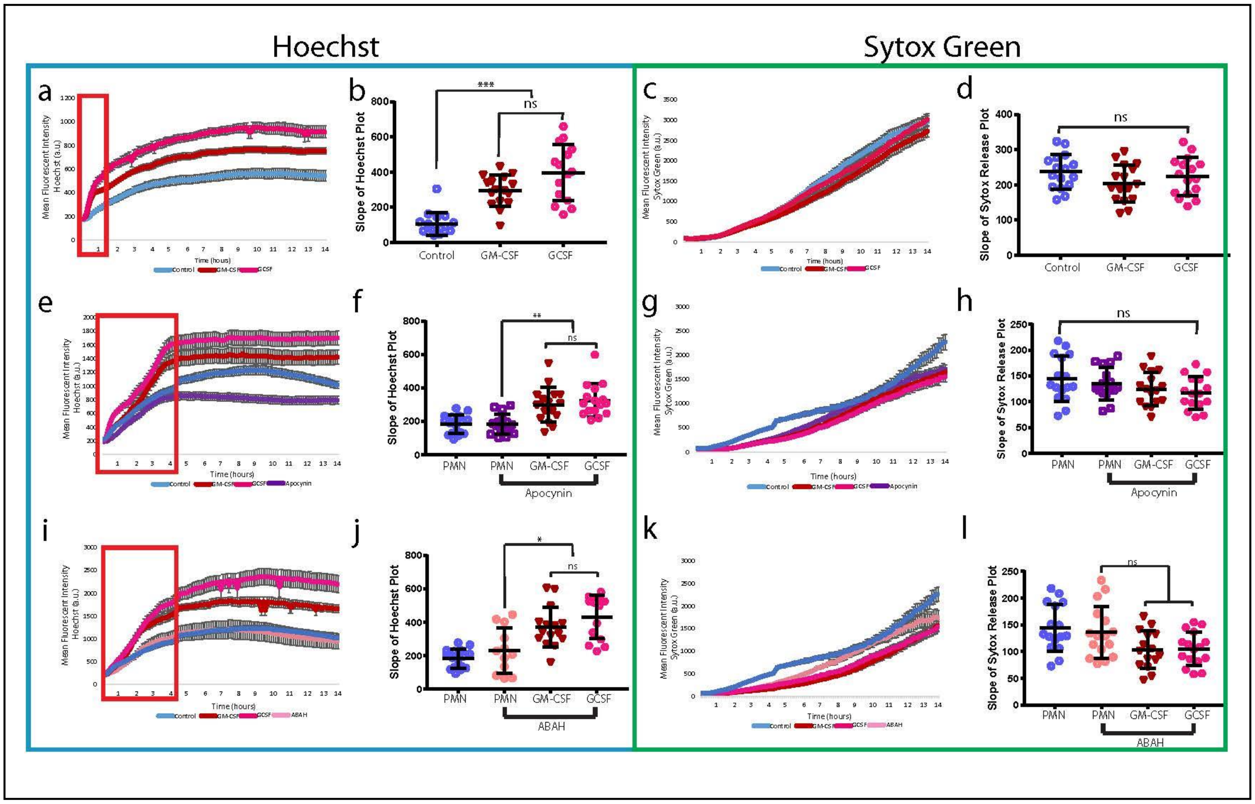

Supplementary Figure 11: GM-CSF or GCSF Boost Swarming but not NET Release Profiles

GM-CSF and GCSF treatments accelerate swarming responses, increase the number of neutrophils, and have minimal impact on NET release. The MFI of Hoechst stained neutrophils was quantified over a 14 hour swarming assay (a, e, i). The slope of each profile over the first hour (b) or 4 hours of the assay (f, j), indicated by a red box, was determined and plotted. Sytox green MFI was quantified over a 14 hour swarming assay (c, g, k). The slope of each profile over the whole 14 hour assay was determined and plotted (d, h, l). N=16 swarms from a representative donor except for GCSF, ABAH GM-CSF for which N=15, ABAH GCSF for which N=14 and ABAH for which N=12 (a-l). n.s. is non-significant, *$p \leq 0.05$ ($p = 0.0117$ for ABAH PMN vs GM-CSF and $p = 0.0002$ ABAH vs GCSF in j), **$p \leq 0.01$ ($p = 0.0015$ for Apocynin PMN vs GM-CSF and $p = 0.0002$ for Apocynin vs GCSF in f,). ***$p \leq 0.001$ ($p = 0.0005$ for control vs GM-CSF and $p < 0.0001$ for control vs GCSF in b). Kruskal-Wallis with Dunn's multiple comparisons test b, f. One way ANOVA with Tukeys post-test d, h, j, l. Error bars represent mean +/- standard deviation (b, d, f, h, j, l) or mean +/- standard error (a, c, e, g, I, k).
