# Supplementary material for: Neutrophil swarming delays the growth of clusters of pathogenic fungi
Source: Nat Commun. 2020 Apr 27;11:2031. doi: 10.1038/s41467-020-15834-4 (PMC7184738; doi:10.1038/s41467-020-15834-4)
Supplement: Supplementary file 2 — Description of Additional Supplementary Files [file 41467_2020_15834_MOESM2_ESM.pdf]

Description of Additional Supplementary Files

File Name: Supplementary Movie 1

Description: **Human Neutrophils Restrict C. albicans Hyphae and Release NETs**. Time-lapse movies showing the containment of C. albicans growth by human neutrophils (left panel) compared to the unchallenged growth of C. albicans (right panel) are shown. This is followed by timelapse imaging showing Hoechst staining (left) and Sytox green staining (right) during swarming. The tight nucleus staining of the Hoechst staining becomes diffuse over time, as Sytox green stains the extracellularly released DNA, demonstrating the release of NETs during neutrophil swarming to C.albicans.

File Name: Supplementary Movie 2

Description: **ROS Inhibitors Disrupt Neutrophil Swarming**. Time-lapse movies are showing swarming to C. albicans (first column). NADPH oxidase inhibition by Apocynin (second column) or DPI (third column) disrupts these dynamics. Apocynin treatment disrupts the early neutrophil recruitment during swarming. DPI treatment results in rapid formation followed by rapid dissolution of the swarms.
