# Supplementary material for: Neutrophil swarming delays the growth of clusters of pathogenic fungi
Source: Nat Commun. 2020 Apr 27;11:2031. doi: 10.1038/s41467-020-15834-4 (PMC7184738; doi:10.1038/s41467-020-15834-4)
Supplement: Supplementary file 5 — Reporting Summary [file 41467_2020_15834_MOESM5_ESM.pdf]

natureresearch

Corresponding author(s): Daniel Irimia

Last updated by author(s): 2020/04/25

Reporting Summary

Nature Research wishes to improve the reproducibility of the work that we publish. This form provides structure for consistency and transparency in reporting. For further information on Nature Research policies, see Authors & Referees and the Editorial Policy Checklist .

Statistics

For all statistical analyses, confirm that the following items are present in the figure legend, table legend, main text, or Methods section.

n/a	Confirmed	
☐	☒	The exact sample size (*n*) for each experimental group/condition, given as a discrete number and unit of measurement
☐	☒	A statement on whether measurements were taken from distinct samples or whether the same sample was measured repeatedly
☐	☒	The statistical test(s) used AND whether they are one- or two-sided<br>*Only common tests should be described solely by name; describe more complex techniques in the Methods section.*
☒	☐	A description of all covariates tested
☐	☒	A description of any assumptions or corrections, such as tests of normality and adjustment for multiple comparisons
☐	☒	A full description of the statistical parameters including central tendency (e.g. means) or other basic estimates (e.g. regression coefficient) AND variation (e.g. standard deviation) or associated estimates of uncertainty (e.g. confidence intervals)
☐	☒	For null hypothesis testing, the test statistic (e.g. *F*, *t*, *r*) with confidence intervals, effect sizes, degrees of freedom and *P* value noted<br>*Give P values as exact values whenever suitable.*
☒	☐	For Bayesian analysis, information on the choice of priors and Markov chain Monte Carlo settings
☒	☐	For hierarchical and complex designs, identification of the appropriate level for tests and full reporting of outcomes
☒	☐	Estimates of effect sizes (e.g. Cohen's *d*, Pearson's *r*), indicating how they were calculated

Our web collection on statistics for biologists contains articles on many of the points above.

Software and code

Policy information about availability of computer code

Data collection	NIS Elements was used in the collection of all timelapse and endpoint images used for work.
Data analysis	NIS Elements (v4.00.12) and FIJI (FIJI Is Just ImageJ, v2.0.0-rc-59/1.52p) were used in the analysis for this work. GraphPad Prism software (v 7.03) was used for statistical analysis.

For manuscripts utilizing custom algorithms or software that are central to the research but not yet described in published literature, software must be made available to editors/reviewers. We strongly encourage code deposition in a community repository (e.g. GitHub). See the Nature Research guidelines for submitting code & software for further information.

Data

Policy information about availability of data

All manuscripts must include a data availability statement. This statement should provide the following information, where applicable:

- Accession codes, unique identifiers, or web links for publicly available datasets
- A list of figures that have associated raw data
- A description of any restrictions on data availability

The source data for Fig 1b-e, 2b-e, 3b-f, 4a-b and 4e-hg, 5a-e, 6a-e, 7a-h and Supplemental Figures 2, 3b-f, 5b-c, 6c-e, 7c-f, 8a-b, 9b-f, 10a-e and 11a-l are provided as a Source Data file. All other data is available from the authors upon request.

Field-specific reporting

Please select the one below that is the best fit for your research. If you are not sure, read the appropriate sections before making your selection.

Life sciences study design

All studies must disclose on these points even when the disclosure is negative.

Sample size	Sample size consisted of N= 3+ donors for all experiments unless noted otherwise. Sample size was below N=3 for experiments solely meant to confirm previously observed and published results (e.g. the role of LTB4 with inhibitors MK-886 and U75302) or those experiments not central to claims of the paper (e.g. swarming to A. fumigatus).
Data exclusions	Data from all experiments were included in analysis, except for two that were excluded. These experiments were excluded due a failure of the device to seal effectively, resulting in leaking from and between the wells.
Replication	To ensure reproducibility, we conducted our replicate experiments on different days, with different donors and with new cultures of fungi each time. Our replicates were consistent. Exact data for each experiment (number of "n", number of independent donors) has been outlined in relevant figure legends and is summarized in Supplementary Table 1.
Randomization	We used multiple donors across experiments and, to our best knowledge, no donor was used more than once. For each experiment, isolated neutrophils were resuspended all together before being randomly aliquoted into experimental treatment groups.
Blinding	Blinding was not used in this study. To protect against bias, manual data analysis was done according to specific and consistent rules. Area analysis was done by outlining the outer edge of the swarm, always including any elements of fungal growth (e.g. protruding hyphae). Intensity analysis was taken over the same area for all groups.

Reporting for specific materials, systems and methods

We require information from authors about some types of materials, experimental systems and methods used in many studies. Here, indicate whether each material, system or method listed is relevant to your study. If you are not sure if a list item applies to your research, read the appropriate section before selecting a response.

Materials & experimental systems

n/a	Involved in the study	
☐	☒	Antibodies
☐	☒	Eukaryotic cell lines
☒	☐	Palaeontology
☒	☐	Animals and other organisms
☐	☒	Human research participants
☒	☐	Clinical data

Methods

n/a	Involved in the study	
☒	☐	ChIP-seq
☒	☐	Flow cytometry
☒	☐	MRI-based neuroimaging

Antibodies

Antibodies used	p47 phox (NCF1) D-10: Santa Cruz Biotechnology, Cat# sc-17845.<br>mAb AC-15 anti–β-actin: Sigma-Aldrich, Cat# A5441<br>Anti-Histone H3 citrulline R2+R8+R17: abcam, Cat# ab5103<br>donkey anti-rabbit IgG Cy3: Jackson Immunoresearch, Cat# 705-165-147
Validation	p47 phox (NCF1) D-10: This antibody is recommended as suitable and validated for western blot on the manufacturer's website, with multiple citations.<br>mAb AC-15 anti–β-actin: This antibody is recommended as suitable and validated for western blot on the manufacturer's website, with multiple citations and a protocol available.<br>Anti-Histone H3 citrulline R2+R8+R17: This antibody is recommended as suitable for immunofluorescence on the manufacturer's website. It has been published in relation to NET staining in multiple publications, including references (56) and (58).<br>donkey anti-rabbit IgG Cy3: This antibody is recommended as suitable for secondary immunofluorescent labeling on the manufacturer's website for use against rabbit derived primary antibodies. This antibody has been used directly with the Anti-Histone H3 citrulline R2+R8+R17" antibody for NET staining in references (56) and (58). A "no primary" antibody control was always run with this secondary to ensure non-specific binding was minimal.

Eukaryotic cell lines

Policy information about cell lines

Cell line source(s)	The ER-HoxB8 cell line is described in reference and maintained in the Mansour lab (59).<br>The NCF-1 ER-HoxB8 lines were generated for this publication, derived from the wild type ER-HoxB8 line.
Authentication	NCF-1 ER-HoxB8 lines were authenticated by western blot to confirm the NCF-1 deficiency and functionally by ROS production assay.

Mycoplasma contamination	Mycoplasma testing was not conducted for the WT and NCF-1 KO ER-HoxB8 lines.
Commonly misidentified lines (See ICLAC register)	N/A

Human research participants

Policy information about studies involving human research participants

Population characteristics	Blood was purchased from Research Blood Components, LLC (Boston, MA). We have no information regarding the characteristics of the donors, except that they were considered healthy donors. We assume they are representative of the general population.
Recruitment	Research Blood Components collects blood based on a research protocol that has been approved by the New England Institutional Review Board (NEIRB), with proper consent from healthy volunteers. Collection of samples from consented patients was approved by the internal review board at the National Insitutes of Health (NIH) under protocol # 93-I-0119 (NCT00001355) and institutional review board at the Massachusetts General Hospital (MGH).
Ethics oversight	Research Blood Components collects blood based on a protocol approved by the New England Institutional Review Board. Protocols involved in this study were approved by the Massachusetts General Hospital institutional review board and by the internal review board at the National Institutes of Health.

Note that full information on the approval of the study protocol must also be provided in the manuscript.
